# Structural diversity in the coordination compounds of cobalt, nickel and copper with *N*-alkylglycinates: crystallographic and ESR study in the solid state†

**DOI:** 10.1039/d1ra04219j

**Published:** 2021-07-06

**Authors:** Darko Vušak, Neven Smrečki, Senada Muratović, Dijana Žilić, Biserka Prugovečki, Dubravka Matković-Čalogović

**Affiliations:** Department of Chemistry, Faculty of Science, University of Zagreb Horvatovac 102a HR-10000 Zagreb Croatia biserka@chem.pmf.hr; Laboratory for Magnetic Resonances, Division of Physical Chemistry, Ruđer Bošković Institute Bijenička 54 HR-10000 Zagreb Croatia

## Abstract

Reactions of *N*-methylglycine (HMeGly), *N*-ethylglycine-hydrochloride (H_2_EtGlyCl) and *N*-propylglycine-hydrochloride (H_2_PrGlyCl) with cobalt(ii), nickel(ii) and copper(ii) ions in aqueous solutions resulted in ten new coordination compounds [Co(MeGly)_2_(H_2_O)_2_] (1), [{Co(MeGly)_2_}_2_(μ-OH)_2_]·2H_2_O (1d), [Cu(MeGly)_2_(H_2_O)_2_] (2α), [Co(EtGly)_2_(H_2_O)_2_] (3), [Ni(EtGly)_2_(H_2_O)_2_] (4), [Cu(μ-EtGly)_2_]_*n*_ (5p), [Co(PrGly)_2_(H_2_O)_2_] (6), [Ni(PrGly)_2_(H_2_O)_2_] (7), and two polymorphs of [Cu(PrGly)_2_(H_2_O)_2_] (8α and 8β). Compounds were characterized by single-crystal and powder X-ray diffraction, infrared spectroscopy, thermal analysis and X-band electron spin resonance (ESR) spectroscopy. These studies revealed a wide range of structural types including monomeric, dimeric and polymeric architectures, as well as different polymorphs. In all monomeric compounds, except 2α, and in the coordination polymer 5p hydrogen bonds interconnect the molecules into 2D layers with the alkyl chain pointing outward of the layer. In 2α and in the dimeric compound 1d hydrogen bonds link the molecules into 3D structures. 1d with cobalt(iii), and 4 and 7 with nickel(ii) are ESR silent. The ESR spectra of 1, 3 and 6 are characteristic for paramagnetic high-spin cobalt(ii). The ESR spectra of all copper(ii) coordination compounds show that the unpaired copper electron is located in the d_*x*^2^−*y*^2^_ orbital, being in agreement with the elongated octahedral geometry.

## Abstract 2

Interactions in copper, nickel and cobalt complexes with *N*-methyl-, *N*-ethyl- and *N*-propylglycinate: monomers, dimer and polymers.

## Graphical abstract

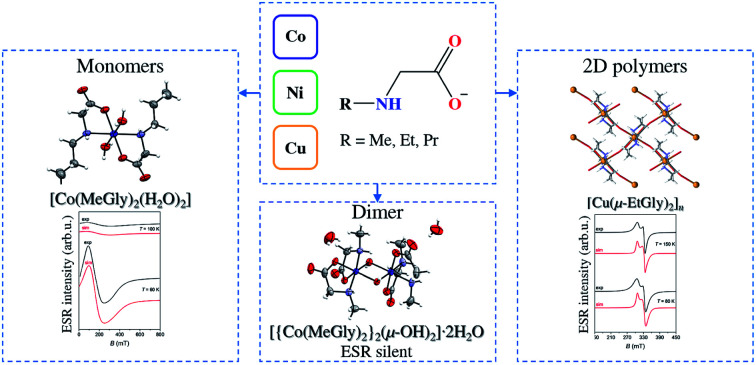

## Introduction

*N*-Alkylated-α-amino acids are present in nature and their biocatalytic properties, as well as chemical syntheses, are widely investigated.^1–3^ They are useful building blocks in peptide science and have found application in structure–activity relationship studies.^4–7^ The simplest modification of an amino acid is by *N*-methylation, so probably the most intensively investigated *N*-alkylated-α-amino acids are *N*-methyl-amino acids, especially *N*-methylglycine (sarcosine), *N*,*N*-dimethylglycine and *N*,*N*,*N*-trimethylglycine (betaine).^8^*N*-Methylation can be useful for conformational studies since introduction of *N*-methyl groups promotes conformational constraints and can also improve pharmacokinetic properties of some peptides.^9–11^*N*-Methylglycine is currently used as a dietary supplement, as a non-specific glycine transport inhibitor, and for treatment of schizophrenia and depression.^12,13^*N*-Ethylglycine acts as an inhibitor of glycine uptake and inhibits pain signaling and is a promising candidate for chronic pain treatment.^14^

Structurally characterized coordination compounds with *N*-alkylated amino acids, especially those containing longer hydrocarbon chains at the amino nitrogen atom are quite rare.^15^ To the best of our knowledge, no coordination compounds with *N*-ethylglycine and *N*-propylglycine have been structurally characterized up to now. There are a few structurally characterized coordination compounds with *N*-methylglycine. It was shown that the *N*-methylglycine moiety can occur in different forms: as an anion, a zwitterion or a cation. In the anionic form, the *N*-methylglycinato moiety acts as a bidentate ligand with *O* and *N* atoms involved in metal coordination.^16–20^ In the case of the cationic and zwitterion forms, it acts as a monodentate, bidentate or bridging ligand.^21–26^ Such coordination compounds may show different magnetic properties, depending on the metal oxidation state, local geometry around the metal center, metal-to-metal separation, bridging ligands, dimensionality of the complexes and non-covalent interacations.

As a part of our ongoing research on synthesis, structural and magnetic characterization as well as biological activity of molybdenum, cobalt, nickel and copper coordination compounds with amino acids, amides and their derivatives^27–39^ we report synthesis, structural and magnetic characterization of ten novel coordination compounds with *N*-alkylglycinates. We have performed the reactions of copper(ii), nickel(ii) and cobalt(ii) compounds with *N*-methylglycine (HMeGly), *N*-ethylglycine-hydrochloride (H_2_EtGlyCl) and *N*-propylglycine-hydrochloride (H_2_PrGlyCl) in aqueous solutions, and characterized the obtained complexes by X-ray diffraction, IR and electron spin resonance (ESR)/electron paramagnetic resonance (EPR) spectroscopy, and thermoanalytical methods (TG/DTA). The following new compounds were obtained: [Co(MeGly)_2_(H_2_O)_2_] (1), [{Co(MeGly)_2_}_2_(μ-OH)_2_]·2H_2_O (1d), [Cu(MeGly)_2_(H_2_O)_2_] (2α), [Co(EtGly)_2_(H_2_O)_2_] (3), [Ni(EtGly)_2_(H_2_O)_2_] (4), [Cu(μ-EtGly)_2_]_*n*_ (5p), [Co(PrGly)_2_(H_2_O)_2_] (6), [Ni(PrGly)_2_(H_2_O)_2_] (7), and two polymorphs of [Cu(PrGly)_2_(H_2_O)_2_] (8α and 8β), where ‘d’ and ‘p’ stand for dimer and polymer, respectively. Our main goal was to study the influence of the alkyl chain length and type of the metal ion on the molecular structure, crystal packing and magnetic properties in the solid state. ESR was used to investigate magnetic properties and to establish magneto-structural correlation in the synthesized compounds.

## Materials and methods

### Materials and physical measurements

All chemicals for the syntheses were purchased from commercial sources (Aldrich, Acros or Alfa Aesar) and used as received without further purification. *N*-Ethylglycine-hydrochloride (H_2_EtGlyCl) and *N*-propylglycine-hydrochloride (H_2_PrGlyCl) were prepared by aminolysis of chloroacetic acid according to the method of E. Fischer (Scheme S1†).^40^ CHN analyses were performed on a Perkin-Elmer 2400 Series II CHNS analyzer in the Analytical Services Laboratory at the Ruđer Bošković Institute, Zagreb, Croatia. The IR spectra were obtained in the range 4000–450 cm^−1^ on a Perkin-Elmer Spectrum Two™ FTIR-spectrometer in ATR mode. The TGA measurements were performed at a heating rate of 10 °C min^−1^ in the temperature range 25–600 °C, under a nitrogen flow of 150 mL min^−1^ on a Mettler-Toledo TG/SDTA 851^e^ instrument. Approximately 10 mg of each sample was placed in a standard aluminum crucible (40 μL).

ESR measurements were conducted on a Bruker Elexsys 580 FT/CW spectrometer. The used microwave frequency was around 9.7 GHz; the magnetic field modulation amplitude was 0.5 mT and the modulation frequency was 100 kHz. Samples were studied in the range from room down to liquid helium temperature.

### Preparation of Co, Ni and Cu coordination compounds with the *N*-methylglycinato ligand

Sodium hydroxide solution (0.08 g, 2 mmol in 10 mL water) was added to an aqueous solution containing *N*-methylglycine (0.53 g, 6 mmol) and corresponding metal acetate (2 mmol in 40 mL water). The mixture was stirred for a few minutes and left to stand at room temperature. Crystals of the coordination compounds [Co(MeGly)_2_(H_2_O)_2_] (1), [{Co(MeGly)_2_}_2_(μ-OH)_2_]·2H_2_O (1d), [Ni(MeGly)_2_(H_2_O)_2_] (1c) and [Cu(MeGly)_2_(H_2_O)_2_] (2α) suitable for X-ray structural analysis, were obtained by slow evaporation of the above-mentioned reaction mixtures. For synthesis of [Cu(μ-MeGly)_2_]_*n*_ (2p), *N*-methylglycine (0.038 g, 0.5 mmol) and Cu(OH)_2_ (0.24 g, 0.25 mmol) were mixed in 10 mL of water. Crystals of [Cu(μ-MeGly)_2_]_*n*_ were obtained by slow evaporation of the solution. The coordination compounds were filtered off and washed with cold water (5 mL). A bulk sample of all compounds (except 1d) was taken for a powder X-ray diffraction experiment in order to confirm their purity. Powder patterns of the compounds were consistent with those calculated from the respective crystal structures (Fig. S1†). Only a few crystals of [{Co(MeGly)_2_}_2_(μ-OH)_2_] (1d) were obtained from the solution that remained after isolation of 1 after several days. [Ni(MeGly)_2_(H_2_O)_2_] (1c) and [Cu(μ-MeGly)_2_]_*n*_ (2p) were prepared by different synthetic methods from the ones described in the literature.^19,20,41^ In the literature, a monoclinic polymorph of [Cu(MeGly)_2_(H_2_O)_2_] (2β), (CSD refcode POBDIT)^17^ was structurally characterized while we have obtained the triclinic polymorph (2α). If 2α is recrystallized from different solvents, it is possible to crystallize either the pure triclinic polymorph 2α, or pure 2p, or a mixture of 2α and 2p, or a mixture of 2p and 2β, as described below.

#### [Co(MeGly)_2_(H_2_O)_2_] (1)

Synthesis as described above, with cobalt(ii) acetate tetrahydrate (0.50 g, 2.0 mmol). Rose-red crystals, yield: 0.30 g (56%). Anal. calc. for C_6_H_16_N_2_O_6_Co (271.14): C 26.58, H 5.95, N 10.33%. Found: C 26.70, H 6.07, N 10.25%. Selected IR(ATR) data (cm^−1^): 3243(s), 3206(s), 3004(w), 2973(w), 2927(w), 2886(w), 2865(w), 2806(w), 1594(vs), 1489(m), 1461(m), 1402(s), 1318(m), 1288(w), 1158(m), 1093(m), 1037(m), 975(m), 950(m), 932(s), 802(w), 734(vs), 647(s), 617(s), 490(m).

#### [{Co(MeGly)_2_}_2_(μ-OH)_2_]·2H_2_O (1d)

Several days after removal of crystals of 1, purple crystals of 1d formed in the solution. Selected IR data (cm^−1^): 3390(m), 3212(m), 3005(w), 2982(w), 2936(w), 2782(w), 2698(w), 1628(m), 1570(vs), 1406(s), 1390(m), 1365(m), 1318(m), 1186(w), 1086(m), 1044(m), 1015(m), 967(m), 936(m), 925(m), 756(w), 647(m), 619(m), 509(w), 460(w).

#### [Ni(MeGly)_2_(H_2_O)_2_] (1c)

Synthesis as described above, with nickel(ii) acetate tetrahydrate (0.50 g, 2.0 mmol). Light blue crystals, yield 0.38 g: (70%). The crystal structure of 1c is already published, however, the compound was synthesized by using a different nickel(ii) salt.^19,41^ Anal. calc. for C_6_H_16_N_2_O_6_Ni (270.90): C 26.60, H 5.95, N 10.34%. Found: C 26.55, H 5.98, N 10.41%. Selected IR data (cm^−1^): 3243(s), 3185(s), 3004(w), 2954(w), 2926(m), 2894(w), 2845(w), 2814(w), 1582(s), 1489(m), 1457(m), 1438(m), 1420(m), 1387(s), 1320(m), 1285(w), 1170(m), 1164(m), 1096(m), 1039(m), 968(m), 924(m), 721(vs), 619(s), 592(s), 506(m).

#### [Cu(MeGly)_2_(H_2_O)_2_] (2α)

Synthesis as described above, with copper(ii) acetate monohydrate (0.40 g, 2.0 mmol). Dark blue crystals, yield: 0.41 (74%). Anal. calc. for C_6_H_16_N_2_O_6_Cu (275.76): C 26.13, H 5.85, N 10.16%. Found: C 26.27, H 5.73, N 10.32%. Selected IR data (cm^−1^): 3363(w), 3242(w), 3190(s), 3017(w), 2983(w), 2964(w), 2936(w), 2809(w), 1612(vs), 1468(m), 1449(m), 1435(s), 1371(s), 1323(m), 1307(m), 1271(m), 1173(m), 1161(m), 1150(m), 1098(m), 1048(m), 983(w), 971(m), 927(s), 743(s), 654(w), 621(s), 496(m), 459(s).

#### [Cu(μ-MeGly)_2_]_*n*_ (2p)

Synthesis as described above, with copper(ii) hydroxide (0.24 g, 0.25 mmol). Dark blue crystals. The crystal structure of 2p is already published, however, the compound was synthesized by using a different copper(ii) compound as the reactant.^20^

#### Recrystallization of [Cu(MeGly)_2_(H_2_O)_2_] (2α)

Recrystallization of 2α from water or a mixture acetone/water (1 : 1 v/v) gave pure 2α. Recrystallization of 2α from a mixture methanol/water (1 : 1 v/v) or from an aqueous solution of ammonium acetate (*c* = 0.1 mol dm^−3^) gave a mixture of 2α and 2p (CSD refcode CORZAM). Recrystallization of 2α from a mixture acetonitrile/water (1 : 1 v/v) gave a mixture of 2p and 2β (CSD refcode POBDIT). Recrystallization of 2α from an aqueous solution of ammonia (*c* = 0.1 mol dm^−3^) gave pure 2p. Powder patterns of crystals obtained after recrystallization are given in Fig. S2.†

### Preparation of Co, Ni and Cu coordination compounds with *N*-ethylglycinato and *N*-propylglycinato ligands

Sodium hydroxide solution (0.16 g, 4 mmol; in 10 mL water) was added to an aqueous solution containing 3 mmol of the ligand (*N*-ethylglycine hydrochloride, 0.42 g, 3 mmol; or *N*-propylglycine hydrochloride, 0.45 g, 3 mmol) and 1 mmol of the corresponding metal acetate in 40 mL water. The mixture was stirred for a few minutes and left to stand at room temperature. The crystals of the coordination compounds [Co(EtGly)_2_(H_2_O)_2_] (3), [Ni(EtGly)_2_(H_2_O)_2_] (4), [Cu(μ-EtGly)_2_]_*n*_ (5p), [Co(PrGly)_2_(H_2_O)_2_] (6), [Cu(PrGly)_2_(H_2_O)_2_] (8α and 8β) suitable for X-ray structural analysis, were obtained by slow evaporation of the above-mentioned reaction mixtures. A bulk sample of each compound was taken for powder X-ray diffraction experiment in order to confirm their purity. It was confirmed that the powder patterns of the synthesized compounds were consistent with powder patterns calculated from the respective crystal structures (Fig. S1†). The two polymorphs 8α and 8β crystallize from the same reaction mixture. In some cases only 8α crystallized from the solution while in others a mixture of the polymorphs crystallized. The two polymorphs could be crystallized separately from 8α in different solvents. The coordination compounds were filtered off and washed with cold water (5 mL). The crystals of 7 were not suitable for single-crystal X-ray structural analysis, however, other analyses (TGA, IR, PXRD) suggest the structural formula [Ni(PrGly)_2_(H_2_O)_2_].

#### [Co(EtGly)_2_(H_2_O)_2_] (3)

Synthesis as described above, with cobalt(ii) acetate tetrahydrate (0.25 g, 1.0 mmol). Rose-red crystals, yield: 0.23 g (77%). Anal. calc. for C_8_H_20_N_2_O_6_Co (299.19): C 32.12, H 6.74, N 9.36%. Found: C 32.40, H 6.74, N 9.37%. Selected IR data (cm^−1^): 3332(s), 3276(s), 2994(w), 2972(w), 2960(w), 2936(w), 2868(w), 1599(vs), 1461(m), 1435(m), 1383(s), 1348(m), 1331(m), 1270(m), 1251(m), 1161(m), 1121(m), 1096(m), 1037(m), 1004(m), 988(m), 921(m), 863(w), 821(w), 737(vs), 600(s), 556(m).

#### [Ni(EtGly)_2_(H_2_O)_2_] (4)

Synthesis as described above, with nickel(ii) acetate tetrahydrate (0.25 g, 1.0 mmol). Light blue crystals, yield: 0.25 g (83%). Anal. calc. for C_8_H_20_N_2_O_6_Ni (298.95): C 32.14, H 6.74, N 9.37%. Found: C 32.27, H 6.66, N 9.28%. Selected IR data (cm^−1^): 3327(s), 3278(s), 2996(w), 2973(w), 2962(w), 2937(w), 2867(w), 1601(vs), 1461(m), 1437(m), 1391(s), 1384(s), 1349(m), 1331(m), 1272(m), 1252(m), 1163(m), 1122(m), 1094(m), 1042(m), 1012(m), 990(m), 924(m), 861(w), 824(w), 810(w), 741(vs), 602(s), 560(s).

#### [Cu(μ-EtGly)_2_]_*n*_ (5p)

Synthesis as described above, with copper(ii) acetate monohydrate (0.20 g, 1.0 mmol). Dark blue crystals, yield: 0.22 g (81%). Anal. calc. for C_8_H_16_N_2_O_4_Cu (267.78): C 35.88, H 6.02, N 10.46%. Found: C 35.75, H 6.13, N 10.68%. Selected IR data (cm^−1^): 3179(s), 2990(w), 2965(w), 2930(w), 2900(w), 2877(w), 2855(w), 1617(vs), 1483(m), 1465(w), 1450(w), 1436(m), 1419(w), 1384(s), 1368(m), 1323(m), 1235(m), 1162(w), 1153(w), 1138(m), 1092(m), 1046(m), 999(m), 984(m), 959(w), 932(s), 897(m), 804(m), 740(s), 622(s), 555(s), 492(s).

#### [Co(PrGly)_2_(H_2_O)_2_] (6)

Synthesis as described above, with cobalt(ii) acetate tetrahydrate (0.25 g, 1.0 mmol). Rose-red crystals, yield: 0.28 g (85%). Anal. calc. for C_10_H_24_N_2_O_6_Co (327.24) C 36.70, H 7.39, N 8.56%. Found: C 36.68, H 7.68, N 8.29%. Selected IR data (cm^−1^): 3335(m), 3221(s), 2965(w), 2952(w), 2927(w), 2870(w), 1697(w), 1593(vs), 1488(m), 1474(m), 1454(w), 1444(w), 1392(s), 1375(s), 1329(m), 1299(m), 1246(m), 1159(m), 1131(m), 1092(m), 1060(m), 1040(m), 1004(m), 951(m), 915(m), 891(w), 785(m), 745(vs), 714(m), 602(s), 549(m), 515(m).

#### [Ni(PrGly)_2_(H_2_O)_2_] (7)

Synthesis as described above, with nickel(ii) acetate tetrahydrate (0.25 g, 1.0 mmol). Light blue powder, yield: 0.30 g (91%). Anal. calc. for C_10_H_24_N_2_O_6_Ni (327.00): C 36.73, H 7.40, N 8.57%. Found: C 36.91, H 7.59, N 8.53%. Selected IR data (cm^−1^): 3290(s), 3248(m), 3141(m), 2971(m), 2958(w), 2948(w), 2927(w), 2878(w), 2866(w), 1604(vs), 1465(m), 1435(m), 1397(s), 1372(s), 1327(m), 1297(m), 1223(m), 1152(m), 1130(m), 984(m), 950(m), 935(m), 890(w), 871(m), 794(w), 747(s), 626(m), 593(s), 561(m), 509(m).

#### [Cu(PrGly)_2_(H_2_O)_2_] (8α)

Synthesis as described above, with copper(ii) acetate monohydrate (0.20 g, 1.0 mmol). Dark blue crystals, yield: 0.29 g (88%). In another synthesis from the same reactants, a mixture of 8α and 8β crystallized from solution. Anal. calc. for C_10_H_24_N_2_O_6_Cu (331.86): C 36.19, H 7.29, N 8.44%. Found (for 8α): C 36.39, H 7.43, N 8.42%. Selected IR data for 8α (cm^−1^): 3448(s), 3391(m), 3214(m), 2974(m), 2960(w), 2938(w), 2879(w), 1634(m), 1600(vs), 1470(m), 1425(m), 1394(s), 1381(s), 1370(s), 1329(m), 1306(m), 1229(m), 1161(m), 1131(m), 1097(m), 1069(m), 1028(w), 985(m), 936(m), 894(w), 877(m), 759(m), 739(m), 604(m), 578(m), 509(s).

Crystallization of the [Cu(PrGly)_2_(H_2_O)_2_] polymorphs starting from 8α. Pure 8β was obtained from water, an aqueous solution of ammonia (*c* = 0.1 mol dm^−3^) or mixtures acetonitrile/water (1 : 1 v/v) and acetone/water (1 : 1 v/v). Recrystallization of 8α from a mixture methanol/water (1 : 1 v/v) gave pure 8α. A mixture of both polymorphs crystallized from an aqueous solution of ammonium acetate (*c* = 0.1 mol dm^−3^). Powder patterns of the crystallization products are given in Fig. S2.†

#### [Cu(PrGly)_2_(H_2_O)_2_] (8β)

Anal. calc. for C_10_H_24_N_2_O_6_Cu (331.86): C 36.19, H 7.29, N 8.44%. Found (for 8β): C 36.25, H 7.47, N 8.56%. Selected IR data for 8β (cm^−1^): 3461(s), 3228(s), 2969(m), 2959(w), 2935(w), 2877(w), 1668(m), 1644(s), 1625(vs), 1472(m), 1452(w), 1427(m), 1386(s), 1360(s), 1327(m), 1307(m), 1290(w), 1228(m), 1161(m), 1132(m), 1096(m), 1078(m), 1029(w), 983(m), 968(m), 935(m), 880(m), 755(m), 742(m), 632(w), 615(w), 601(w), 584(m), 511(m).

### X-ray diffraction analysis

Single-crystal X-ray diffraction data of the coordination compounds were collected by ω-scans on an Oxford Diffraction Xcalibur3 CCD diffractometer with graphite-monochromated MoKα radiation. Room temperature single-crystal X-ray diffraction data for 2p, 3 and 4 were collected on an XtaLAB Synergy-S diffractometer with CuKα radiation. Data reduction was performed using the CrysAlis software package.^42^ Solution, refinement and analysis of the structures were done using the programs integrated in the WinGX system.^43^ All structures were solved by direct methods (SHELXS) and by dual-space methods (SHELXT), and the refinement procedure was performed by the full-matrix least-squares method based on *F*^2^ against all reflections using SHELXL.^44–46^ The non-hydrogen atoms were refined anisotropically. All hydrogen atoms were located in the difference Fourier maps. Because of poor geometry for some of them, they were placed in calculated positions and refined using the riding model. Hydrogen atoms of the coordinated and crystallization water molecules were found in difference Fourier maps and the O–H distances were fixed to 0.85(1) Å, and the H–H distances were fixed to 1.39(2) Å. Geometrical calculations were done using PLATON.^47^ Drawings of the structures were prepared using PLATON and MERCURY programs.^48^ The crystallographic data are summarized in Tables S1–S3.† Based on the crystal structures of polymorphs 8α and 8β as well as monoclinic (2β), (CSD refcode POBDIT) and triclinic (2α) polymorph of [Cu(MeGly)_2_(H_2_O)_2_] the Hirshfeld surface was generated using program *CrystalExplorer17*.^49^ Additionally, Hirshfeld surface fingerprint plots were generated representing 2D histograms of the *d*_i_ and *d*_e_ distances; *d*_i_ corresponds to the distance from a point on the surface to the nearest nucleus inside the surface and *d*_e_ corresponds to the distance from a point on the surface to the nearest nucleus outside the surface.^50^ Powder X-ray diffraction (PXRD) data were collected on a Malvern Panalytical Aeris powder diffractometer in the Bragg–Brentano geometry with PIXcel^1D^ detector, using CuKα radiation (*λ* = 1.5406 Å). Samples were contained on a Si sample holder. Powder patterns were collected at room temperature in the range from 5° to 30°(2*θ*) with a step size of 0.043° and 7.14 s per step. The data were collected and visualized by using the Malvern Panalytical HighScore Software Suite.^51^

#### Crystal data for 1

C_6_H_16_CoN_2_O_6_, *M* = 271.14, monoclinic, space group *C*2/*c* (no. 15), *a* = 16.8488(8), *b* = 9.3909(4), *c* = 6.8017(3) Å, *β* = 97.788(5)°, *V* = 1066.28(8) Å^3^, *T* = 295 K, *Z* = 4, *D*_calc_ = 1.689 g cm^−3^, *μ*(MoKα) = 1.622 mm^−1^, 3485 reflections measured, 3485 unique (*R*_int_ = 0.033). The final *R*_1_(*F*, *I* > 2*σ*(*I*)) value was 0.0264, w*R*_2_(*F*^2^, *I* > 2*σ*(*I*)) = 0.0780, *S* = 0.96. CCDC 2069284.†

#### Crystal data for 1d

C_12_H_30_Co_2_N_4_O_12_, *M* = 540.26, orthorhombic, space group *Pbca* (no. 61), *a* = 15.5085(8), *b* = 14.6844(6), *c* = 18.0637(9) Å, *V* = 4113.7(3) Å^3^, *T* = 295 K, *Z* = 8, *D*_calc_ = 1.745 g cm^−3^, *μ*(MoKα) = 1.681 mm^−1^, 18 569 reflections measured, 4042 unique (*R*_int_ = 0.074). The final *R*_1_(*F*, *I* > 2*σ*(*I*)) value was 0.0424, w*R*_2_(*F*^2^, *I* > 2*σ*(*I*)) = 0.0909, *S* = 1.02. CCDC 2069285.†

#### Crystal data for 2α

C_6_H_16_CuN_2_O_6_, *M* = 275.76, triclinic, space group *P*1̄ (no. 2), *a* = 7.0311(10), *b* = 7.4825(11), *c* = 10.6021(10) Å, *α* = 78.541(10)°, *β* = 89.938(10)°, *γ* = 78.408(13)°, *V* = 535.07(12) Å^3^, *T* = 295 K, *Z* = 2, *D*_calc_ = 1.712 g cm^−3^, *μ*(MoKα) = 2.053 mm^−1^, 4095 reflections measured, 2321 unique (*R*_int_ = 0.020). The final *R*_1_(*F*, *I* > 2*σ*(*I*)) value was 0.0292, w*R*_2_(*F*^2^, *I* > 2*σ*(*I*)) = 0.082, *S* = 1.09. CCDC 2069291.†

#### Crystal data for 2p

C_6_H_12_CuN_2_O_4_, *M* = 239.72, monoclinic, space group *P*2_1_/*c* (no. 14), *a* = 7.9367(1), *b* = 5.9953(1), *c* = 9.0214(2) Å, *β* = 90.522(2)°, *V* = 429.247(13) Å^3^, *T* = 295 K, *Z* = 2, *D*_calc_ = 1.855 g cm^−3^, *μ*(CuKα) = 3.531 mm^−1^, 10 596 reflections measured, 876 unique (*R*_int_ = 0.034). The final *R*_1_(*F*, *I* > 2*σ*(*I*)) value was 0.0303, w*R*_2_(*F*^2^, *I* > 2*σ*(*I*)) = 0.0907, *S* = 1.09. CCDC 2084505.†

#### Crystal data for 3

C_8_H_20_CoN_2_O_6_, *M* = 299.19, triclinic, space group *P*1̄ (no. 2), *a* = 5.4524(2), *b* = 7.1733(3), *c* = 8.7708(3) Å, *α* = 113.139(4)°, *β* = 91.989(3)°, *γ* = 106.635(3)°, *V* = 298.00(2) Å^3^, *T* = 150 K, *Z* = 1, *D*_calc_ = 1.667 g cm^−3^, *μ*(MoKα) = 1.459 mm^−1^, 4798 reflections measured, 1298 unique (*R*_int_ = 0.030). The final *R*_1_(*F*, *I* > 2*σ*(*I*)) value was 0.0230, w*R*_2_(*F*^2^, *I* > 2*σ*(*I*)) = 0.0583, *S* = 1.07. CCDC 2069290.†

#### Crystal data for 3 at room temperature

C_8_H_20_CoN_2_O_6_, *M* = 299.19, triclinic, space group *P*1̄ (no. 2), *a* = 5.4717(3), *b* = 7.1941(4), *c* = 8.8468(4) Å, *α* = 113.095(4)°, *β* = 92.008(4)°, *γ* = 106.385(5)°, *V* = 303.11(3) Å^3^, *T* = 295 K, *Z* = 1, *D*_calc_ = 1.639 g cm^−3^, *μ*(CuKα) = 11.317 mm^−1^, 3012 reflections measured, 1186 unique (*R*_int_ = 0.051). The final *R*_1_(*F*, *I* > 2*σ*(*I*)) value was 0.0484, w*R*_2_(*F*^2^, *I* > 2*σ*(*I*)) = 0.01261, *S* = 1.06. CCDC 2084504.†

#### Crystal data for 4

C_8_H_20_N_2_NiO_6_, *M* = 298.95, triclinic, space group *P*1̄ (no. 2), *a* = 5.4231(3), *b* = 7.1382(4), *c* = 8.7608(4) Å, *α* = 113.806(5)°, *β* = 91.508(4)°, *γ* = 106.771(5)°, *V* = 293.12(3) Å^3^, *T* = 150 K, *Z* = 1, *D*_calc_ = 1.694 g cm^−3^, *μ*(MoKα) = 1.675 mm^−1^, 4697 reflections measured, 1272 unique (*R*_int_ = 0.022). The final *R*_1_(*F*, *I* > 2*σ*(*I*)) value was 0.0175, w*R*_2_(*F*^2^, *I* > 2*σ*(*I*)) = 0.0430, *S* = 1.09. CCDC 2069289.†

#### Crystal data for 4 at room temperature

C_8_H_20_N_2_NiO_6_, *M* = 298.95, triclinic, space group *P*1̄ (no. 2), *a* = 5.44882(8), *b* = 7.15455(10), *c* = 8.83552(11) Å, *α* = 113.6920(12)°, *β* = 91.6006(11)°, *γ* = 106.5605(12)°, *V* = 298.320(8) Å^3^, *T* = 295 K, *Z* = 1, *D*_calc_ = 1.664 g cm^−3^, *μ*(CuKα) = 2.568 mm^−1^, 7113 reflections measured, 1268 unique (*R*_int_ = 0.023). The final *R*_1_(*F*, *I* > 2*σ*(*I*)) value was 0.0221, w*R*_2_(*F*^2^, *I* > 2*σ*(*I*)) = 0.0599, *S* = 1.11. CCDC 2084506.†

#### Crystal data for 5p

C_8_H_16_CuN_2_O_4_, *M* = 267.77, monoclinic, space group *P*2_1_/*c* (no. 14), *a* = 10.0467(8), *b* = 6.3568(5), *c* = 9.0286(9) Å, *β* = 114.398(10)°, *V* = 525.12(9) Å^3^, *T* = 295 K, *Z* = 2, *D*_calc_ = 1.694 g cm^−3^, *μ*(MoKα) = 2.076 mm^−1^, 4536 reflections measured, 1074 unique (*R*_int_ = 0.024). The final *R*_1_(*F*, *I* > 2*σ*(*I*)) value was 0.0277, w*R*_2_(*F*^2^, *I* > 2*σ*(*I*)) = 0.0703, *S* = 1.05. CCDC 2069288.†

#### Crystal data for 6

C_10_H_24_CoN_2_O_6_, *M* = 327.24, monoclinic, space group *P*2_1_/*c* (no. 14), *a* = 10.4296(13), *b* = 7.2609(9), *c* = 10.0987(10) Å, *β* = 107.734(13)°, *V* = 728.42(16) Å^3^, *T* = 295 K, *Z* = 2, *D*_calc_ = 1.492 g cm^−3^, *μ*(MoKα) = 1.201 mm^−1^, 2294 reflections measured, 2294 unique (*R*_int_ = 0.051). The final *R*_1_(*F*, *I* > 2*σ*(*I*)) value was 0.0379, w*R*_2_(*F*^2^, *I* > 2*σ*(*I*)) = 0.0823, *S* = 0.86. CCDC 2069287.†

#### Crystal data for 8α

C_10_H_24_CuN_2_O_6_, *M* = 331.85, monoclinic, space group *I*2/*a* (no. 15), *a* = 9.3725(4), *b* = 7.3714(4), *c* = 21.2849(7) Å, *β* = 90.496(3)°, *V* = 1470.49(11) Å^3^, *T* = 295 K, *Z* = 4, *D*_calc_ = 1.499 g cm^−3^, *μ*(MoKα) = 1.508 mm^−1^, 2865 reflections measured, 1597 unique (*R*_int_ = 0.023). The final *R*_1_(*F*, *I* > 2*σ*(*I*)) value was 0.0379, w*R*_2_(*F*^2^, *I* > 2*σ*(*I*)) = 0.0809, *S* = 1.07. CCDC 2069286.†

#### Crystal data for 8β

C_10_H_24_CuN_2_O_6_, *M* = 331.85, monoclinic, space group *P*2_1_/*c* (no. 14), *a* = 11.5413(6), *b* = 11.2713(5), *c* = 5.8447(2) Å, *β* = 93.037(4)°, *V* = 759.24(6) Å^3^, *T* = 295 K, *Z* = 2, *D*_calc_ = 1.452 g cm^−3^, *μ*(MoKα) = 1.460 mm^−1^, 7331 reflections measured, 2003 unique (*R*_int_ = 0.027). The final *R*_1_(*F*, *I* > 2*σ*(*I*)) value was 0.0298, w*R*_2_(*F*^2^, *I* > 2*σ*(*I*)) = 0.0835, *S* = 1.07. CCDC 2069292.†

## Results and discussion

### Synthesis and thermal properties of the coordination compounds

**Scheme 1:**
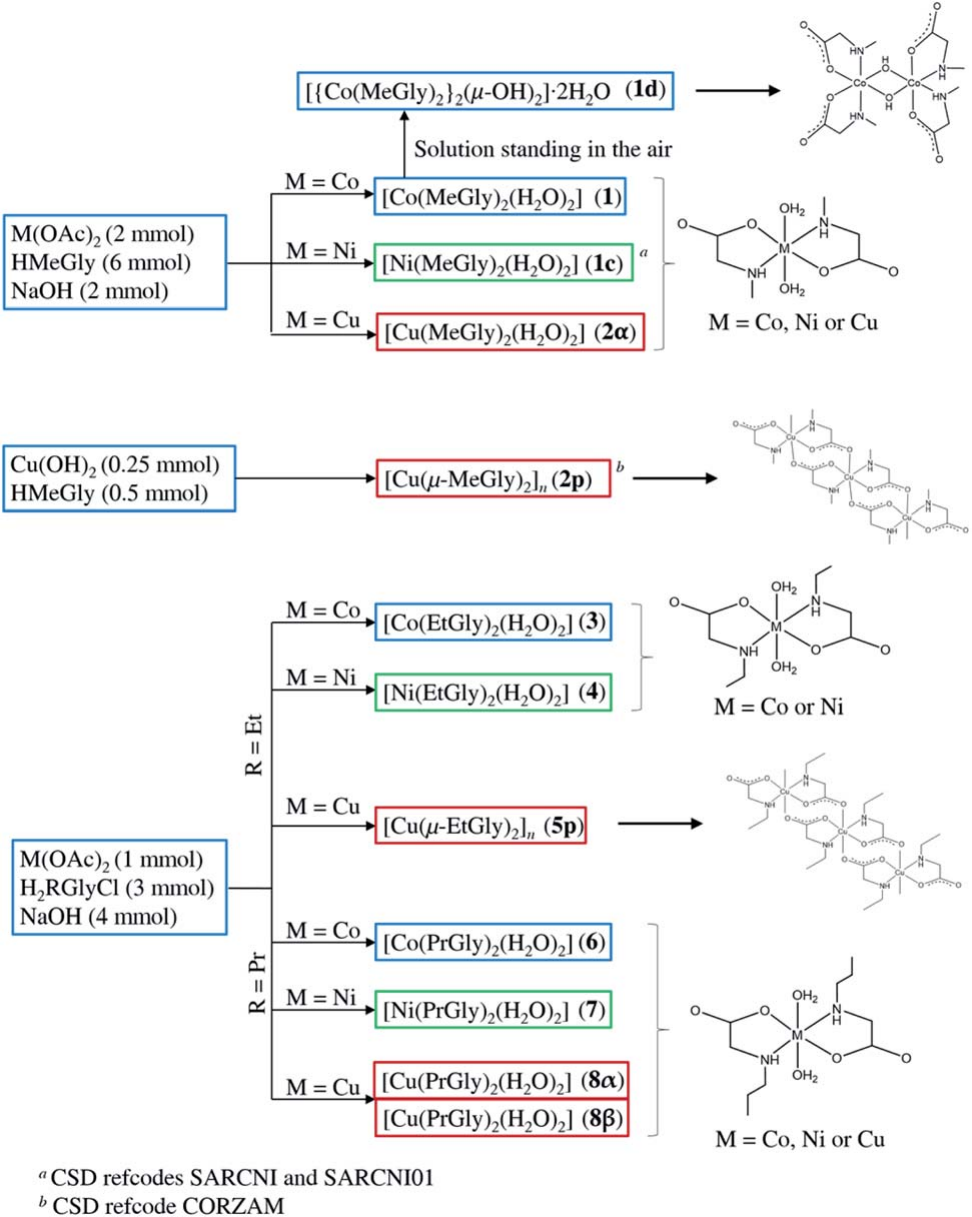
Preparation of the *N*-alkylglycinato coordination compounds by reactions of the ligand (*N*-methylglycine, HMeGly; *N*-ethylglycine-hydrochloride, H_2_EtGlyCl; *N*-propylglycine-hydrochloride, H_2_PrGlyCl) with the metal (Co, Ni and Cu) compounds in aqueous solutions. [Ni(MeGly)_2_(H_2_O)_2_] and [Cu(μ-MeGly)_2_]_*n*_ were synthesized by different procedures and published in CSD by other groups.^19,20,41^ Drawings of each type of complexes are also shown.

All three divalent metal ions formed neutral coordination compounds containing two *N*,*O*-bidentate *N*-alkylglycinato ligands per metal ion (Scheme 1). Cobalt(ii) and nickel(ii) gave analogous monomeric coordination compounds of the general formula [M(RGly)_2_(H_2_O)_2_] (M = Co, Ni; R = methyl, ethyl, or propyl) with yields increasing with the elongation of the hydrocarbon chain R, probably due to their lower solubility. Copper(ii), on the other hand, gave monomeric coordination compounds of the type [Cu(RGly)_2_(H_2_O)_2_] when R = methyl and propyl, and polymeric coordination compounds of the type [Cu(RGly)_2_]_*n*_, R = methyl and ethyl. Since two different copper(ii) compounds were obtained with *N*-methylglycine (monomer and polymer), compound 2α was recrystallized from different solutions to test the stability of each compound under different crystallization conditions. Depending on the conditions, recrystallization of 2α gave either pure 2α, pure 2p, or binary mixtures: 2α and 2p or 2p and 2β (Table 1 and Fig. S2†). Two polymorphs of the copper(ii) coordination compounds with the *N*-propylglycinato ligand were obtained, 8α and 8β. The same synthetic procedures gave in some cases pure 8α, while in others simultaneous appearance of 8α and 8β. Recrystallization of 8α in some conditions gave pure 8α, in some cases pure 8β, and in one case a mixture of the two polymorphs (Table 1 and Fig. S2†). Colour and habitus of the crystals of both polymorphs are very similar so it is not possible to distinguish between them by visual inspection.

**Table 1:** Products of crystallization from different solvents with 2α and 8α as the initial compounds

Solvent/solution	Product of crystallization, starting from 2α	Product of crystallization, starting from 8α
H_2_O	2α	8β
CH_3_OH/H_2_O (1 : 1 v/v)	2α + 2p a	8α
CH_3_CN/H_2_O (1 : 1 v/v)	2p a + 2β b	8β
(CH_3_)_2_CO/H_2_O (1 : 1 v/v)	2α	8β
NH_4_OAc(aq)	2α + 2p a	8α + 8β
NH_3_(aq)	2p a	8β

a CSD refcode CORZAM, [Cu(μ-MeGly)_2_]_*n*_.

b CSD refcode POBDIT, monoclinic polymorph of [Cu(MeGly)_2_(H_2_O)_2_].

Oxidation of cobalt(ii) to cobalt(iii) occurred upon standing of the solution of [Co(MeGly)_2_(H_2_O)_2_] (1) in air, resulting in the formation of the dimeric coordination compound [{Co(MeGly)_2_}_2_(μ-OH)_2_]·2H_2_O (1d). This was possibly the consequence of a considerably greater solubility (lower yield) of 1 in comparison with the analogous cobalt(ii) coordination compounds 3 and 6, since no such by-products were obtained in these cases. Only the aqueous solution of 1 is air-sensitive, while crystals of both 1 and 1d were stable even after standing in the air for several months.

Thermal stability of all monomeric coordination compounds was evaluated by the initial loss of both coordinated water molecules. Nickel(ii) coordination compounds, which dehydrate in the range 90–140 °C, are the most stable, while copper(ii) complexes lose coordinated water molecules at much lower temperatures (90–100 °C). Cobalt(ii) coordination compounds lose coordinated water molecules in the range 90–110 °C. Further decomposition of the dehydrated coordination compounds proceeds with carbonization. The lowest decomposition temperature is observed in copper(ii) coordination compounds (decomposition starts at *ca.* 200–210 °C), while their cobalt(ii) and nickel(ii) analogues (after dehydration) have similar thermal stabilities (decomposition starts at *ca.* 300–320 °C). Full thermal analysis data are given in Table S4.†

Infrared spectra of the coordination compounds were characterized by the presence of very strong and sharp bands of the antisymmetric and symmetric stretching of the carboxylate ion, *ν*_as_(COO) occurring in the range of 1620–1580 cm^−1^, and *ν*_s_(COO) occurring in the range of 1400–1380 cm^−1^. The difference between *ν*_as_(COO) and *ν*_s_(COO) is generally greater than 200 cm^−1^ indicating monodentate coordination mode of the carboxylate ion, as confirmed by the results of the X-ray analysis.^52–54^

A sharp band of medium intensity, which was assigned as O–H stretching, *ν*(OH, H_2_O), was observed in the range 3240–3461 cm^−1^ in the spectra of all monomeric coordination compounds. Comparing the spectra of the monomeric cobalt(ii), nickel(ii) and copper(ii) compounds, the *ν*(OH) bands occur at the highest wavenumbers in the spectra of the copper(ii) compounds. This difference indicates a slightly larger decrease in the O–H bond strength upon coordination to cobalt(ii) and nickel(ii) as compared to copper(ii), possibly due to electron transfer from the O–H to the O–M bond, which would imply stronger coordinative Co–O and Ni–O bonds as compared to Cu–O. On the other hand, the *ν*(NH) bands, observed in the range 3180–3290 cm^−1^, occur at the highest wave numbers in the spectra of cobalt(ii) and nickel(ii) coordination compounds, indicating stronger N–M bonds in the case of the copper(ii) coordination compounds. Bond strengths of M–O and M–N bonds are in accordance with bond lengths obtained from crystallographic data (Tables S5–S7†).

### Crystal structures of monomeric coordination compounds

**Fig. 1:**
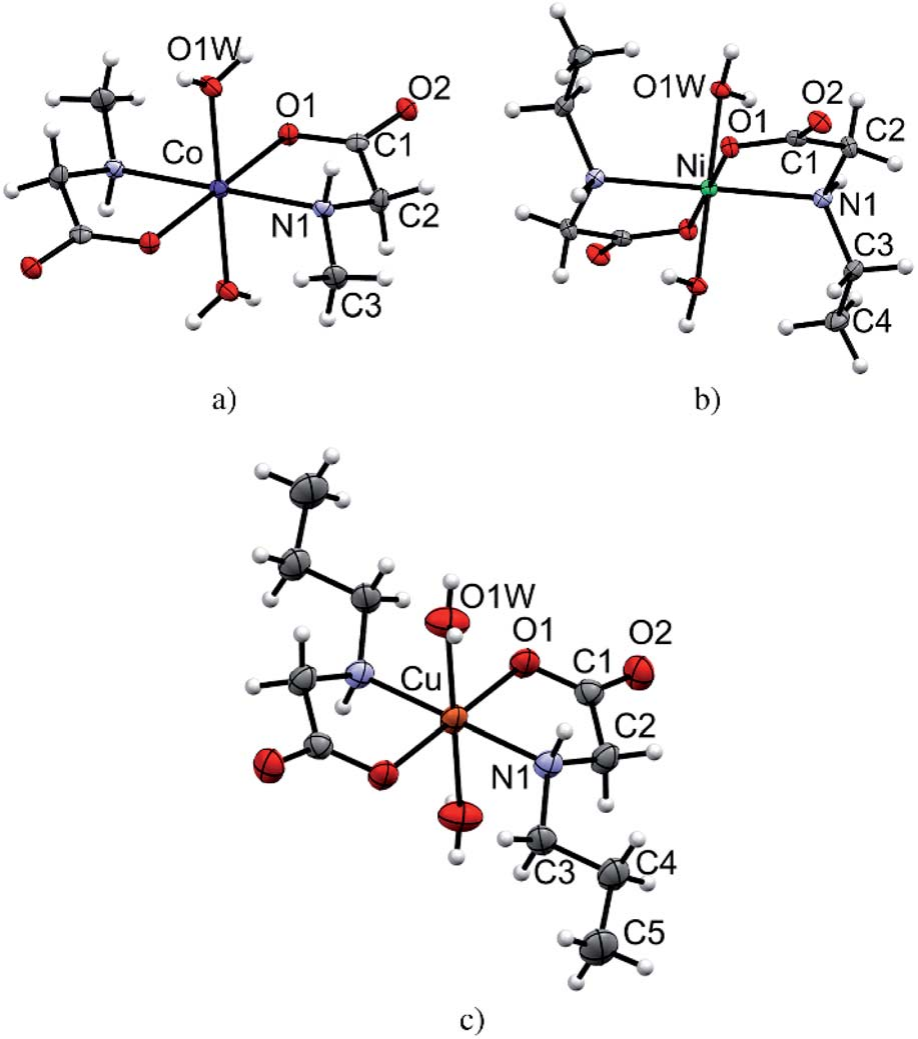
Molecular structures: (a) 1, (b) 4 and (c) 8α as representatives of the monomeric *N*-methylglycinato (1), *N*-ethylglycinato (4) and *N*-propylglycinato (8α) compounds with the atom labeling scheme. Displacement ellipsoids of non-hydrogen atoms are drawn at the 50% probability level.

All monomeric compounds (1, 2α, 3, 4, 6, 8α and 8β) are centrosymmetric with the metal atom lying on the inversion center (detailed crystallographic data are given in Tables S1–S3†). The asymmetric units contain half of the coordination compound molecule, except in 2α where there are two independent halves of molecules. ORTEP plots of one representative molecular structure of a coordination compound with each *N*-alkylglycinato ligand: *N*-methylglycinato (1), *N*-ethylglycinato (4) and *N*-propylglycinato (8α), are presented in Fig. 1. Coordination compounds 2α, 3, 6, and 8β have analogous labeling schemes of the *N*-alkylglycinato ligands as the ones shown (Fig. S3†). Cobalt(ii) and nickel(ii) coordination compounds with *N*-ethylglycinato ligands (3 and 4) are isostructural (Table S2†). We were not able to obtain single-crystals of 7 of good quality to solve the crystal structure, however, thermal analysis, as well as infrared spectroscopy suggests that the molecular structure of 7 is equivalent to that of 6, 8α and 8β.

The metal atom in the structures of all monomeric compounds (1, 2α, 3, 4, 6, 8α and 8β) is octahedrally coordinated by two *N*,*O*-bidentate *N*-alkylglycinato ligands in the equatorial positions and two water molecules occupying the axial coordination sites (Fig. 1 and S3†). The amino nitrogen atoms are arranged in the *trans*-position. The copper(ii) ion in compounds 2α, 8α and 8β exhibits the typical Jahn–Teller distorted [4 + 2] coordination geometry. The longer axial bonds are toward the coordinated water molecules (Table S5†).

**Fig. 2:**
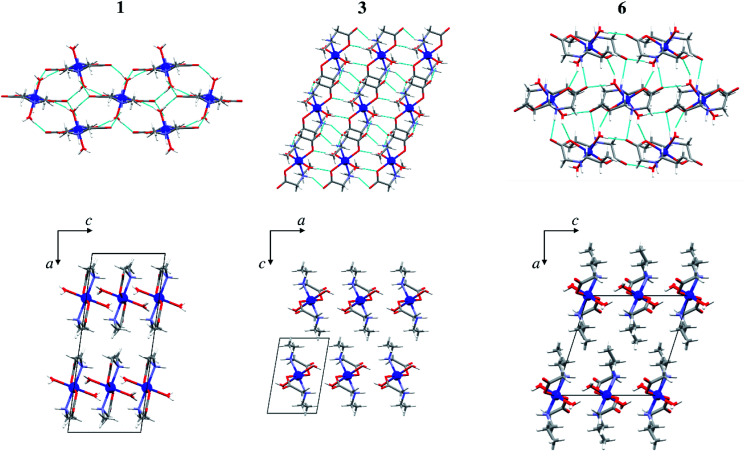
Hydrogen bonds forming 2D layers in 1, 3 and 6 (upper row), and packing of layers in 1, 3 and 6 parallel to (010) (lower row). Hydrogen bonds are shown as light blue lines.

In all monomeric compounds, except 2α, hydrogen bonds interconnect the molecules into 2D layers (Fig. 2a). All metal atoms within the hydrogen-bonded 2D layer are coplanar. Although the alkyl chains are of different lengths (methyl in 1, ethyl in 3 and 4, and propyl in 6, 8α and 8β) the hydrogen bond motif within the layer is the same in all compounds except 8β which has two additional hydrogen bonds. In all monomeric compounds the hydrogen atom from the amino nitrogen atom N1 serves as a hydrogen bond donor to the carboxylate oxygen atom O2 which is not coordinated to the metal atom. The shortest N⋯O hydrogen bond, *d*(N1⋯O2) = 2.970(3) Å, is in compound 1 (Table S8†). Additionally, the coordinated water molecule O1W is a hydrogen bond donor to both carboxylate oxygen atoms O1 and O2 with the shortest hydrogen bond length *d*(O1W⋯O2) of 2.721(3) Å in 1 (Table S8†). Fig. 2 (upper row) shows hydrogen bonds forming 2D layers in compounds 1, 3 and 6 as a representative of the monomeric compounds. The alkyl chains in 1, 3 and 6 and in all monomeric compounds, except 2α, point outward of the 2D layers forming only weak van der Waals contacts (Fig. 2, lower row). Geometries of the intermolecular hydrogen bonds are given in Table S8.†

**Fig. 3:**
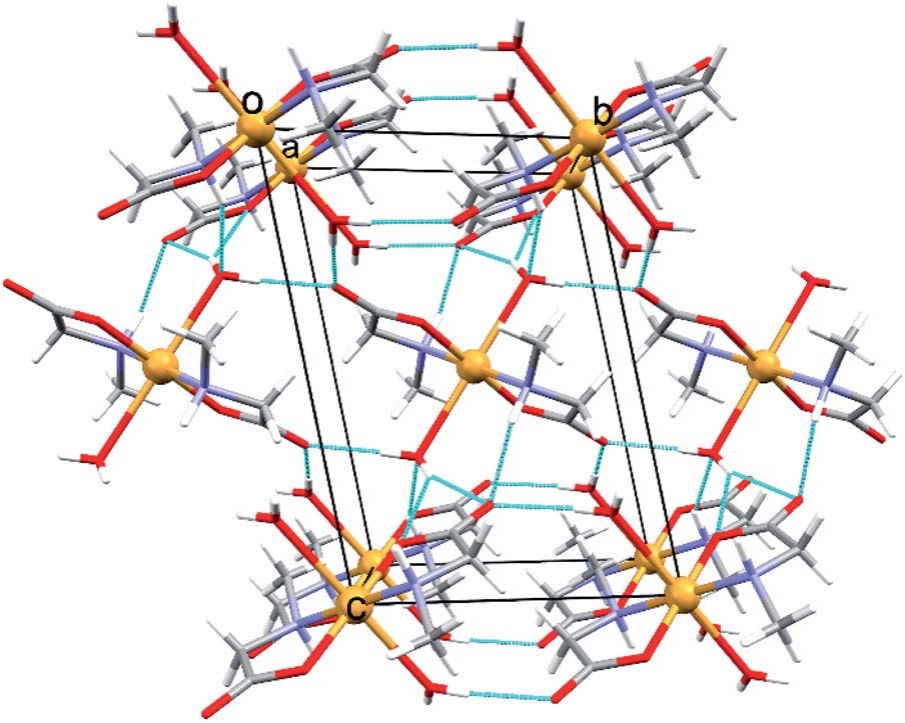
Perspective view of the crystal packing with hydrogen bonds in 2α along the crystallographic axis [100]. Hydrogen bonds are shown as light blue lines.

In the triclinic polymorph of [Cu(MeGly)_2_(H_2_O)_2_] (2α) (two independent halves of the molecules in the asymmetric unit) hydrogen bonds link the molecules into a 3D structure (Fig. 3). Coordinated water molecules, as well as amino nitrogen atoms, serve as hydrogen bond donors to the carboxylate oxygen atoms, both between symmetrically dependent and independent molecules. The shortest hydrogen bond length is that between the symmetrically dependent molecules and involves the oxygen atom from the coordinated water molecule (O1W) and the carboxylate oxygen atom O21 which is not coordinated to the copper atom (Table S8†). Two 2D layers of hydrogen bonds forming a 3D supramolecular structure are shown in Fig. S4.† The monoclinic polymorph of [Cu(MeGly)_2_(H_2_O)_2_] (2β) also forms a 3D supramolecular structure. The main structural difference between the two polymorphs is the orientation of the water molecule in the complex molecule (Fig. S5†). As a consequence, the two polymorphs have slightly different intermolecular contacts. Hirshfeld surfaces and fingerprint plots showing intermolecular contacts are given in Fig. S6.†

**Fig. 4:**
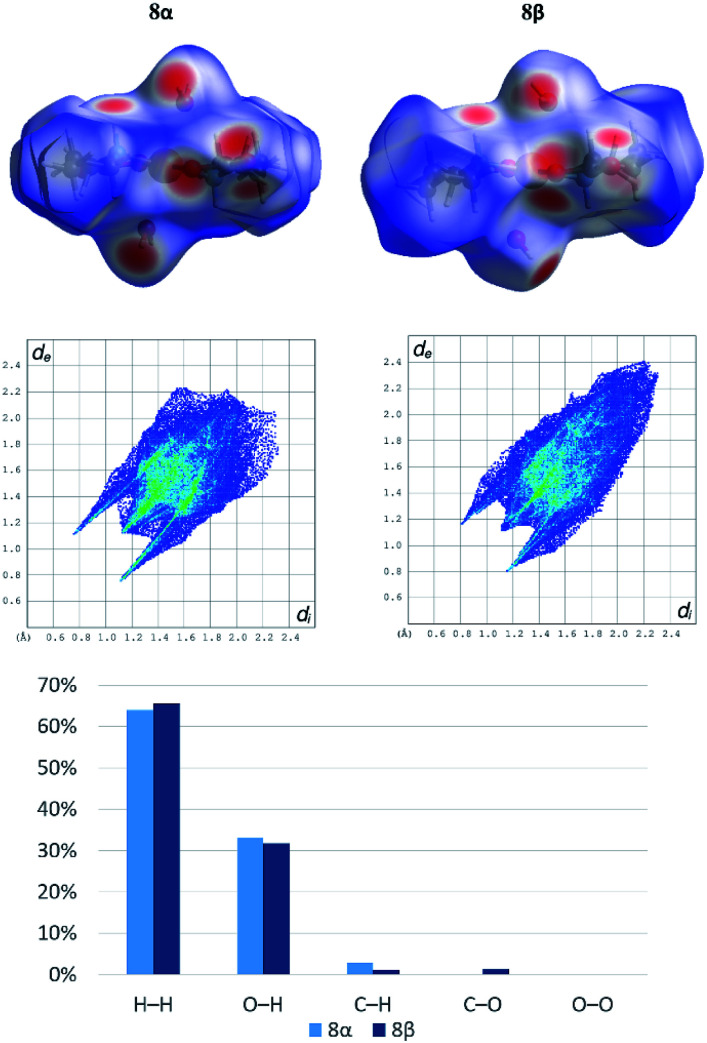
Hirshfeld fingerprint plot with decomposition of the dominant types of intermolecular contacts in 8α and 8β: H–H (64.0% in 8α; 65.6% in 8β), O–H (33.0% in 8α; 31.7% in 8β), C–H (2.8% in 8α; 1.1% in 8β), C–O (0% in 8α; 1.4% in 8β), and O–O (0.2% in 8α; 0.2% in 8β).

Both polymorphs [Cu(PrGly)_2_(H_2_O)_2_] (8α and 8β) crystallize in the monoclinic crystal system but with different unit cell parameters and space groups (8α in *I*2/*a* and 8β in *P*2_1_/*c*, see Table S3†). There is only a small difference in the molecular conformation of 8α and 8β, mostly in the orientation of the coordinated water molecules (Fig. S7†). However, this small difference has a significant impact on the crystal packing. In the crystal structure of 8β there are two additional bifurcated hydrogen bonds. Amino nitrogen atom connects two molecules through the N–H⋯O_carboxylate_ hydrogen bond and the hydrogen bond involving the coordinated water molecule toward carboxylate oxygen atoms of two neighbouring molecules (Fig. S8†). The difference in the hydrogen bonding between two neighbouring complex molecules in polymorphs can be described by graph-set notation of hydrogen bond motifs.^55^ In 8α two rings are formed – *R*^2^_2_(8) and *R*^2^_2_(10), and in 8β there are five rings formed by six hydrogen bonds – 2*R*^1^_2_(6), 2*R*^2^_1_(4) and *R*^2^_2_(8) (Fig. S8†). Non-covalent interactions in the crystal structures of the polymorphs were further investigated by Hirshfeld surface analysis. The 2D fingerprint plots with the decomposition of the dominant types of intermolecular contacts in 8α and 8β are presented in Fig. 4.

Both polymorphs exhibit a pair of long sharp spikes with short *d*_i_ and *d*_e_ values (bottom left of the plot. The upper associated with the donor atom, the lower one with the acceptor) representing the O_water_–H⋯O_carboxylate_ hydrogen bonds. There is also a close C–O_carboxylate_⋯C–O_carboxylate_ contact (Fig. S9†) in 8β (*d*(O⋯C) = 2.970(2) Å), which is characteristic for *trans*-(aminocarboxylato)copper(ii) polymeric coordination compounds.^37^

### Crystal structure of the dimeric compound 1d

**Fig. 5:**
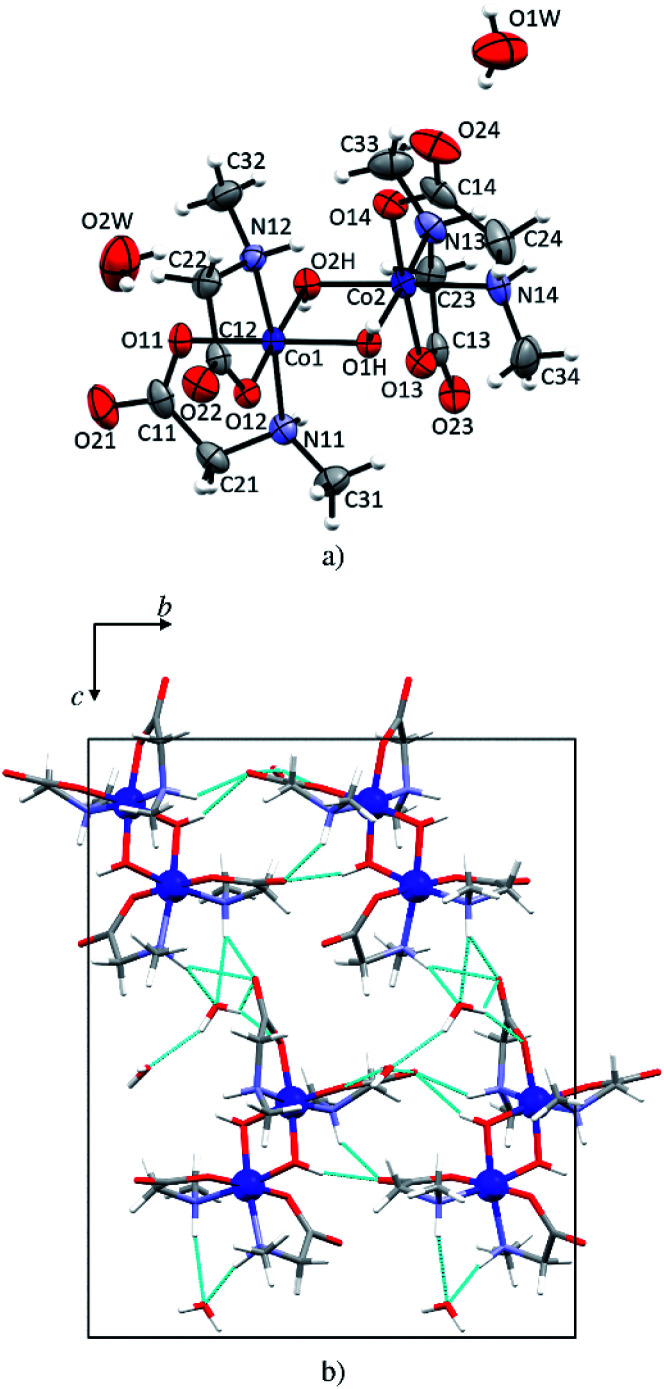
(a) Molecular structure of the dimeric compound 1d with the atom labeling scheme. Displacement ellipsoids of non-hydrogen atoms are drawn at the 50% probability level. (b) One layer parallel to (100). Hydrogen bonds are shown as light blue lines.

In 1d each cobalt(iii) atom is octahedrally coordinated by two *N*-methylglycinato ligands and two hydroxyl groups forming a distorted octahedron (Fig. 5a). This structure is a dihydrate, the only one among the investigated compounds. The structure is dimeric with two hydroxyl groups linking two cobalt(iii) atoms. Such coordination is typical for cobalt(iii) coordination compounds with amino carboxylates, that is, glycinate,^56,57^ alaninate,^58^ valinate,^59^ arginine,^60^ and prolinate.^61^ Co–O (1.886(2)–1.904(3) Å, Table S6†) and Co–N bonds (1.951(3) and 1.973(2) Å, Table S6†) in 1d are shorter than M–O (1.9645(12)–2.162(3) Å, Table S5†) and M–N (2.0043(15)–2.175(3) Å, Table S5†) in the monomeric compounds. The crystal structure is stabilized by an extensive hydrogen-bonding network. Both water molecules of crystallization are involved in hydrogen bonding forming 2D layers (Fig. 5b) but only one water molecule (O1W) is involved in the linkage between the layers thus forming a 3D network. Hydrogen bonds in 1d are given in Table S9.†

### Crystal structure of the copper 2D coordination polymer 5p

**Fig. 6:**
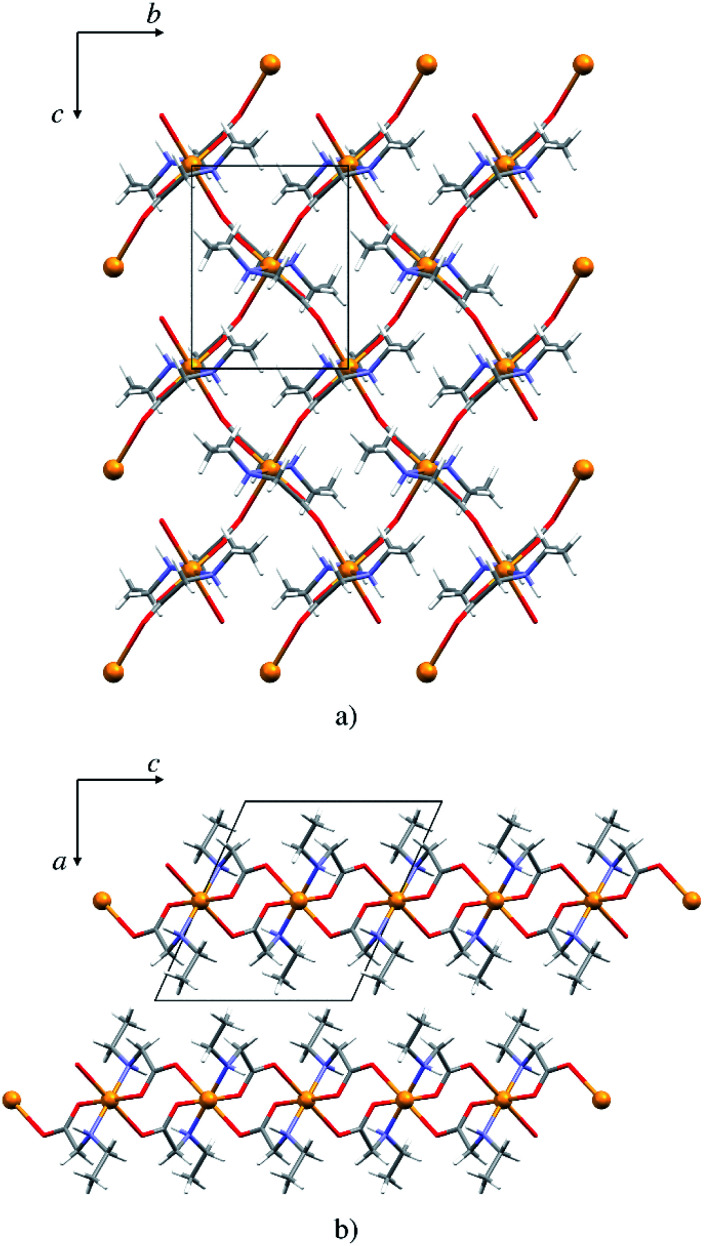
(a) 2D polymeric sheet of 5p parallel to (100), (b) packing of layers in 5p parallel to (010).

The copper atom in 5p is coordinated by two *N*-ethylglycinato ligands in the equatorial plane and the axial coordination sites are occupied by carboxylate oxygen atoms from the neighbouring complex units (Fig. 6). The copper(ii) ion exhibits the Jahn–Teller distorted coordination geometry with four shorter equatorial bonds to the nitrogen and carboxylate oxygen atoms of two *N*-alkylglycinato anions, and the longer axial bonds to the carboxylate oxygen atoms of neighbouring complexes (Table S7†).

The packing is similar as that in the monomeric structures. 2D layers are formed with the alkyl chain pointing outward of the layer, however here the molecules are interlinked by covalent bonds (Fig. 6). Only one type of hydrogen bond is present in the structure, the intermolecular N1–H⋯O1 hydrogen bond (Fig. S10 and Table S9†). Each complex is involved in four hydrogen bond chains (two as hydrogen bond donors and two as acceptors) propagating in two dimensions (Fig. S10†).

### Influence of the alkyl chain on crystal packing

Packing index (PI) was calculated for all structurally characterized complexes and for compounds published in CSD (Table S10†). *N*-Ethylglycinate complexes with formulae [M(EtGly)_2_(H_2_O)_2_] (M = Co, Ni) are most efficiently packed (PI = 75.0%, 74.9% for 3 and 4, respectively), while the *N*-propylglycinate complexes pack least efficiently (PI = 70.7%, 69.2% and 67.4% for 6, 8α and 8β, respectively). In the polymeric copper compounds, 2p is more efficiently packed (PI = 74.9%) than compound 5p (PI = 72.8%). Since only Co complexes of the formulae [Co(RGly)_2_(H_2_O)_2_] (R = methyl, ethyl, or propyl) were obtained with all three *N*-alkylglycinates, these complexes were studied in more detail. The distance between hydrogen bonded layers is shortest for the *N*-ethylglycinate complex 3 (8.03 Å), being in accordance with the efficient packing, and longest for *N*-propylglycinate complex 6 (9.93 Å), while for the *N*-methylglycinate coordination compound 1 it is slightly greater than in 3 (8.35 Å) (Fig. S11†). This result may be surprising, however, *N*-ethylglycinate ligand has larger conformational freedom than *N*-methylglycinate, which allows it to fold in a more efficient way. On the other hand, *N*-propylglycinate with an extra CH_2_ group is large enough to form interpenetrated alkyl chains between the hydrogen bonded layers, thus significantly increasing the interlayer distance (Fig. 2, lower row).

### ESR study

Local magnetic properties of 1, 1d, 2α, 2p, 3, 4, 5p, 6, 7, 8α and 8β coordination compounds were studied by X-band ESR spectroscopy. The oxidation state of the metal centers was confirmed by electron spin resonance (ESR)/electron paramagnetic resonance (EPR) spectroscopy.

**Fig. 7:**
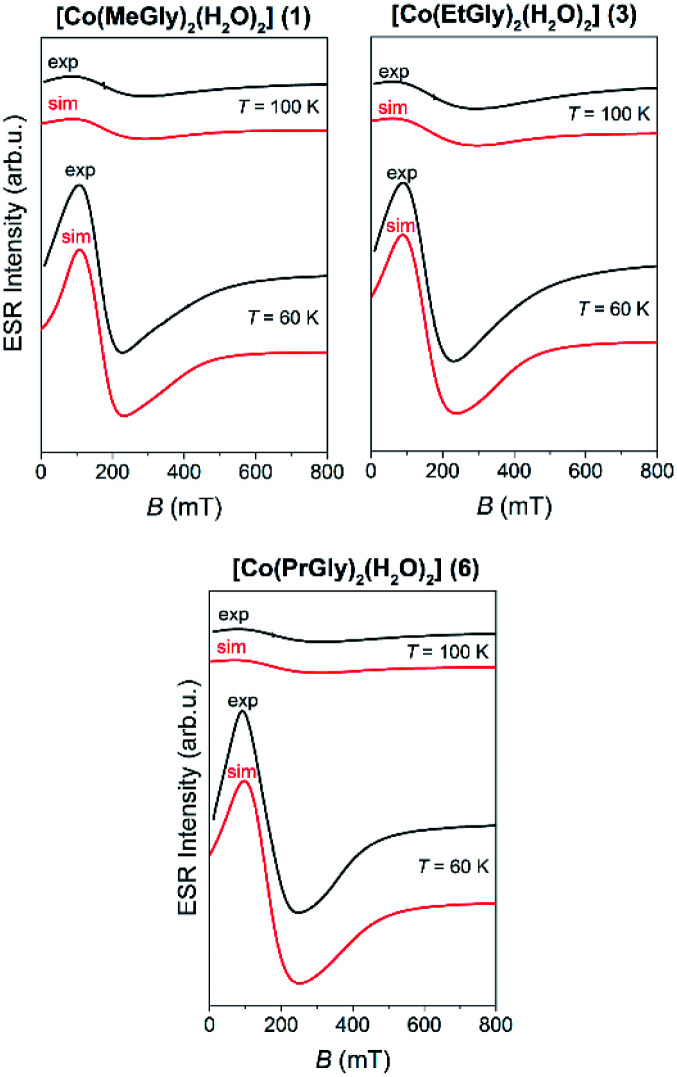
The experimental (black lines) and simulated (red lines) ESR spectra of the Co(ii) coordination compounds (1, 3 and 6) at the indicated temperatures.

The nickel(ii) coordination compounds were ESR silent within the measured temperature range, as it is usually the case for nickel(ii) (non-Kramer's system with *S* = 1).^62^ The dimer coordination compound 1d was also ESR silent in the whole temperature range as expected for coupled integer spins of cobalt(iii) ions.^62^ The cobalt(ii) coordination compounds had no signal at room temperature but after lowering the temperature below 100 K, the signals appeared. The recorded spectra of these coordination compounds at two selected temperatures are shown in Fig. 7.

The recorded spectra are characteristic for paramagnetic high-spin cobalt(ii) ions (*S* = 3/2, d^7^). Octahedral cobalt(ii) ion usually has large zero-field splitting that results with only the lowest states (*m*_s_ = ±1/2) thermally occupied, thus only one ESR line is observed with highly anisotropic *g*-values.^34,62,63^ No hyperfine interaction between electron spin *S* = 1/2 and nuclear spin *I* = 7/2 for cobalt(ii) ions was detected.^64^ Therefore, the following reduced form of spin-Hamiltonian was assumed:

1 *H* = *μ*_B_*BgS*

In eqn (1), the constant *μ*_B_ is Bohr magneton, *B* is external magnetic field, *g* is *g*-tensor, *S* is electron spin operator for the effective cobalt spin of *S* = 1/2. The spectra were simulated by using EasySpin software.^65^ The obtained *g*-values and parameters used for the simulation of cobalt(ii) coordination compounds are given in Table 2 while the simulated spectra are shown in Fig. 7. The same parameters were used for the simulations at different temperatures while only line-width of the used Lorentzian lines were changed with temperature. *g*-Strain parameters were used as factors for line-broadening to obtain better agreement with the experimental spectra.

**Table 2:** The values of spin-Hamiltonian parameters obtained from the spectral simulations of Co(ii) coordination compounds

Compound	*g*-Tensor	*g*-Strain	*l*_w_ (mT)	*T* (K)
1	[6.5 4.0 2.1]	[0 0 0.7]	140	100
		[0 0 0.9]	70	60
3	[7.3 4.5 2.2]	[0 0 0]	180	100
		[0 0 0.8]	95	60
6	[6.5 4.3 2.1]	[0 0 0]	180	100
		[0 0 0.8]	100	60

**Fig. 8:**
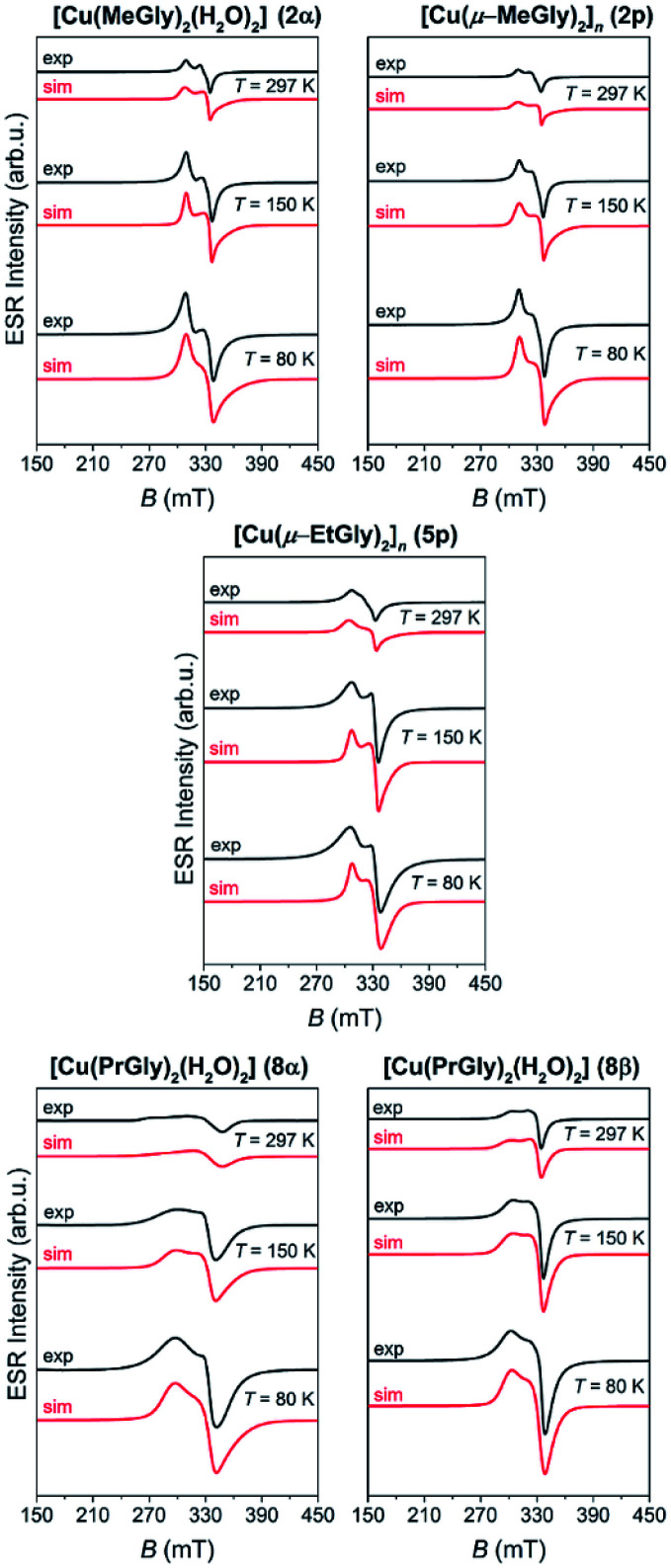
The experimental (black lines) and simulated (red lines) ESR spectra of the copper(ii) coordination compounds at the indicated temperatures.

The representative ESR spectra of the investigated copper(ii) coordination compounds, obtained at the selected temperatures, are shown in Fig. 8. Hyperfine interaction between electron spin *S* = 1/2 and nuclear spins *I* = 3/2 was not detected and therefore the form of spin-Hamiltonian (1) was used for the simulation.^65^ The simulated spectra are shown in Fig. 8, while the parameters used for the simulations are given in Table 3. As was mentioned before for the cobalt coordination compounds, the spectra were simulated taking into consideration only the temperature change of line-width of assumed Lorentzian lines.

**Table 3:** The values of spin-Hamiltonian parameters obtained from the spectral simulations of copper(ii) coordination compounds

Compound	*g*-Tensor	*g*-Strain	*l*_w_ (mT)	*T* (K)
2α	[2.00 2.06 2.23]	[0.18 0 0.07]	2	297
		[0.2 0 0.03]	2	150
		[0.3 0 0.04]	4	80
2p	[2.01 2.06 2.22]	[0.2 0 0.08]	1	297
		[0.2 0 0.06]	2	150
		[0.22 0 0.04]	3	80
5p	[2.03 2.07 2.25]	[0.31 0.006 0.09]	2	297
		[0.15 0 0.05]	3	150
		[0.13 0 0.01]	6	80
8α	[2.00 2.06 2.34]	[0.1 0.12 0.4]	10	297
		[0.21 0 0.17]	7	150
		[0.28 0 0.15]	8	80
8β	[2.06 2.07 2.30]	[0.13 0.01 0.12]	3	297
		[0.15 0 0.15]	4	150
		[0.17 0.03 0.12]	5	80

Although 2p and 5p are coordination polymers, simulations show that their magnetic structures are monomer-like and similar to those of 2α, 8α and 8β. This is due to the fact that the closest Cu⋯Cu distance in the polymeric chain is 5.4 Å and 5.5 Å in 2p and 5p, respectively. From the obtained *g*-values, given in Table 3, one can see that *g*_*x*_ ≈ *g*_*y*_ < *g*_*z*_ for all copper complexes so the unpaired copper electron is located in the d_*x*^2^−*y*^2^_ orbital. This is in agreement with the elongated octahedral copper geometry where *g*_*z*_ is in the direction of the axial distortion.^66^

## Conclusions

Structural diversity was found to depend both on the metal ion and chain length. Cobalt(ii) and nickel(ii) coordination compounds are monomers of the general formula [M(RGly)_2_(H_2_O)_2_] (M = Co, Ni; R = methyl, ethyl, or propyl). Copper(ii) gave monomeric coordination compounds of the type [Cu(RGly)_2_(H_2_O)_2_] when R = methyl and propyl, and also polymeric coordination compounds of the type [Cu(RGly)_2_]_*n*_, R = methyl and ethyl. Two polymorphs of the copper(ii) coordination compound with the *N*-propylglycinato ligand were obtained, 8α and 8β, with significant differences in non-covalent interactions due to the orientation of the coordinated water molecule. Conditions for obtaining pure forms were found by varying solvents. In all monomeric compounds, except copper(ii) with *N*-methylglycinate, hydrogen bonds interconnect the molecules into 2D layers. Although the alkyl chain in the monomers is of different length the hydrogen bond motif within the layers is the same in all compounds except 8β which has two additional hydrogen bonds. In copper(ii) with *N*-methylglycinate the hydrogen bonds link the molecules into a 3D structure. Oxidation of cobalt(ii) to cobalt(iii) occurred upon standing of the solution of monomeric 1 in air, resulting in the formation of 1d with dimeric molecules linked into a 3D structure. 5p is a coordination polymer with 2D layers similar to those in the monomeric compounds. The effect of the alkyl chain length in the cobalt(ii) and nickel(ii) compounds is seen in the efficiency of crystal packing: monomeric *N*-ethylglycinato complexes pack most efficiently.

ESR spectroscopy shows that cobalt(iii) and nickel(ii) coordination compounds are ESR silent. Cobalt(ii) coordination compounds have ESR spectra characteristic for paramagnetic high-spin cobalt(ii) ions (*S* = 3/2, d^7^). ESR spectra of copper(ii) coordination compounds show that the unpaired copper electron is located in the d_*x*^2^−*y*^2^_ orbital, being in agreement with the elongated octahedral coordination in all copper(ii) coordination compounds. Spectra of the polymeric coordination compounds 2p and 5p are similar to those of the monomeric copper(ii) coordination compounds due to large Cu(ii)⋯Cu(ii) distances in these polymers and therefore weak spin–spin interactions between them.

## Conflicts of interest

There are no conflicts to declare.

## Supplementary Material

RA-011-D1RA04219J-s001

RA-011-D1RA04219J-s002

## References

[cit1] Hyslop J. F., Lovelock S. L., Watson A. J. B., Sutton P. W., Roiban G.-D. (2019). J. Biotechnol..

[cit2] Sagan S., Karoyan P., Lequin O., Chassaing G., Lavielle S. (2004). Curr. Med. Chem..

[cit3] Mindt M., Hannibal S., Heuser M., Risse J. M., Sasikumar K., Nampoothiri K. M., Wendisch V. F. (2019). Front. Bioeng. Biotechnol..

[cit4] Patterson A. W., Peltier H. M., Ellman J. A. (2008). J. Org. Chem..

[cit5] Mas-Moruno C., Rechenmacher F., Kessler H. (2010). Anticancer. Agents Med. Chem..

[cit6] Pinalli R., Brancatelli G., Pedrini A., Menozzi D., Hernández D., Ballester P., Geremia S., Dalcanale E. (2016). J. Am. Chem. Soc..

[cit7] Alessandri I., Biavardi E., Gianoncelli A., Bergese P., Dalcanale E. (2016). ACS Appl. Mater. Interfaces.

[cit8] Aurelio L., Brownlee R. T. C., Hughes A. B. (2004). Chem. Rev..

[cit9] Tonelli A. E. (1976). Biopolymers.

[cit10] Manavalan P., Momany F. A. (1980). Biopolymers.

[cit11] Haviv F., Fitzpatrick T. D., Swenson R. E., Nichols C. J., Mort N. A., Bush E. N., Diaz G., Bammert G., Nguyen A. (1993). J. Med. Chem..

[cit12] Lin C.-Y., Liang S.-Y., Chang Y.-C., Ting S.-Y., Kao C.-L., Wu Y.-H., Tsai G. E., Lane H.-Y. (2017). World J. Biol. Psychiatry.

[cit13] Mathew S. J. (2013). Biol. Psychiatry.

[cit14] Werdehausen R., Mittnacht S., Bee L. A., Minett M. S., Armbruster A., Bauer I., Wood J. N., Hermanns H., Eulenburg V. (2015). Pain.

[cit15] Groom C. R., Bruno I. J., Lightfoot M. P., Ward S. C. (2016). Acta Crystallogr., Sect. B: Struct. Sci., Cryst. Eng. Mater..

[cit16] Sabo T. J., Đinović V. M., Kaluđerović G. N., Stanojković T. P., Bogdanović G. A., Juranić Z. D. (2005). Inorg. Chim. Acta.

[cit17] Krishnakumar R. V., Natarajan S., Bahadur S. A., Cameron T. S. (1994). Zeitschrift für Krist. - Cryst. Mater..

[cit18] Larsen S., Watson K. J., Sargeson A. M., Turnbull K. R. (1968). Chem. Commun..

[cit19] Fałtynowicz H., Daszkiewicz M., Wysokiński R., Adach A., Cieślak-Golonka M. (2015). Struct. Chem..

[cit20] Butcher R. J., Brewer G., Zemba M. (2014). Acta Crystallogr., Sect. E: Struct. Rep. Online.

[cit21] Krishnakumar R. V., Subha Nandhini M., Natarajan S. (2001). Acta Crystallogr., Sect. E: Struct. Rep. Online.

[cit22] Gawryszewska P. P., Jerzykiewicz L., Sobota P., Legendziewicz J. (2000). J. Alloys Compd..

[cit23] Ashida T., Bando S., Kakudo M. (1972). Acta Crystallogr., Sect. B: Struct. Crystallogr. Cryst. Chem..

[cit24] Trzebiatowska-Gusowska M., Gągor A., Baran J., Drozd M. (2009). J. Raman Spectrosc..

[cit25] Silva M. R., Beja A. M., Paixäo J. A., da Veiga L. A. (2001). Z. Kristallogr. - New Cryst. Struct..

[cit26] Fleck M., Ghazaryan V. V., Petrosyan A. M. (2013). Acta Crystallogr., Sect. C: Cryst. Struct. Commun..

[cit27] Vušak D., Prugovečki B., Milić D., Marković M., Petković I., Kralj M., Matković-Čalogović D. (2017). Cryst. Growth Des..

[cit28] Prugovečki B., Vušak D., Smrečki N., Kralj M., Uzelac L., Matković-Čalogović D. (2019). Acta Crystallogr., Sect. A: Found. Adv..

[cit29] Tašner M., Prugovečki B., Mrvoš-Sermek D., Korpar-Čolig B., Giester G., Matković-Čalogović D. (2008). Acta Chim. Slov..

[cit30] Tašner M., Prugovečki B., Soldin Ž., Prugovečki S., Rukavina L., Matković-Čalogović D. (2013). Polyhedron.

[cit31] Smokrović K., Muratović S., Karadeniz B., Užarević K., Žilić D., Đilović I. (2020). Cryst. Growth Des..

[cit32] Vušak D., Prugovečki B., Matković-Čalogović D. (2018). Acta Crystallogr., Sect. A: Found. Adv..

[cit33] Vušak D., Ležaić K., Prugovečki B., Matković-Čalogović D. (2019). Acta Crystallogr., Sect. A: Found. Adv..

[cit34] Vušak D., Smrečki N., Prugovečki B., Đilović I., Kirasić I., Žilić D., Muratović S., Matković-Čalogović D. (2019). RSC Adv..

[cit35] Prugovečki B., Vušak D., Smrečki N., Matković-Čalogović D. (2018). Acta Crystallogr., Sect. A: Found. Adv..

[cit36] Pejić J., Vušak D., Szalontai G., Prugovečki B., Mrvoš-Sermek D., Matković-Čalogović D., Sabolović J. (2018). Cryst. Growth Des..

[cit37] Vušak D., Pejić J., Jurković M., Szalontai G., Sabolović J. (2020). CrystEngComm.

[cit38] Matković-Čalogovic D., Vušak D., Smrečki N., Prugovečki B. (2019). Acta Crystallogr., Sect. A: Found. Adv..

[cit39] Smokrović K., Đilović I., Matković-Čalogović D. (2020). CrystEngComm.

[cit40] Fischer E. (1903). Ber. Dtsch. Chem. Ges..

[cit41] Guha S. (1973). Acta Crystallogr., Sect. B: Struct. Crystallogr. Cryst. Chem..

[cit42] CrysAlisPRO Software System, Oxford Diffraction/Agilent Technologies UK Ltd, Yarnto, England, 2018

[cit43] Farrugia L. J. (2012). J. Appl. Crystallogr..

[cit44] Sheldrick G. M. (2008). Acta Crystallogr., Sect. A: Found. Crystallogr..

[cit45] Sheldrick G. M. (2015). Acta Crystallogr., Sect. C: Struct. Chem..

[cit46] Sheldrick G. M. (2015). Acta Crystallogr., Sect. A: Found. Adv..

[cit47] Spek A. L. (2009). Acta Crystallogr., Sect. D: Biol. Crystallogr..

[cit48] Macrae C. F., Bruno I. J., Chisholm J. A., Edgington P. R., McCabe P., Pidcock E., Rodriguez-Monge L., Taylor R., van de Streek J., Wood P. A. (2008). J. Appl. Crystallogr..

[cit49] Turner M. , McKinnon J. J., Wolff S. K., Grimwood D. J., Spackman P. R., Jayatilaka D. and Spackman M. A., CrystalExplorer17, University of Western Australia, 2017

[cit50] Spackman M. A., Jayatilaka D. (2009). CrystEngComm.

[cit51] Degen T., Sadki M., Bron E., König U., Nénert G. (2014). Powder Diffr..

[cit52] Silverstein R. M., Bassler G. C. (1962). J. Chem. Educ..

[cit53] Nakamoto K. , Infrared and Raman Spectra of Inorganic and Coordination Compounds, John Wiley & Sons, Inc., Hoboken, NJ, USA, 2008

[cit54] Deacon G. (1980). Coord. Chem. Rev..

[cit55] Etter M. C. (1990). Acc. Chem. Res..

[cit56] Hamada K., Ohta E., Fujiwara T., Ama T. (1989). Bull. Chem. Soc. Jpn..

[cit57] Versiane O., Rodrigues B. L., Ramos J. M., Téllez C. A., Felcman J. (2006). Spectrochim. Acta, Part A.

[cit58] Sahin O., Büyükgüngör O., Köse D. A., Nefcefoglu H. (2008). Anal. Sci.: X-Ray Struct. Anal. Online.

[cit59] Galán-Mascarós J. R., Martí-Gastaldo C., Murcia-Martínez A. (2008). Solid State Sci..

[cit60] Radivojša P. N., Juranić N., Ćelap M. B., Toriumi K., Saito K. (1991). Polyhedron.

[cit61] Prikhod’ko A., Pointillart F., Golhen S., Gavrilenko K. S., Ouahab L., Kolotilov S. V. (2012). New J. Chem..

[cit62] Carrington A. and McLachlan A. D., Introduction to Magnetic Resonance, Harper & Row, New York, 1967

[cit63] Cockle S. A., Lindskog S., Grell E. (1974). Biochem. J..

[cit64] Damjanović V., Kuzman D., Vrdoljak V., Muratović S., Žilić D., Stilinović V., Cindrić M. (2019). Cryst. Growth Des..

[cit65] Stoll S., Schweiger A. (2006). J. Magn. Reson..

[cit66] Garribba E., Micera G. (2006). J. Chem. Educ..

