# Supplementary material for: Structural diversity in the coordination compounds of cobalt, nickel and copper with N-alkylglycinates: crystallographic and ESR study in the solid state
Source: RSC Adv. 2021 Jul 6;11(38):23779–90. doi: 10.1039/d1ra04219j (PMC9036651; doi:10.1039/d1ra04219j)
Supplement: RA-011-D1RA04219J-s001 [file RA-011-D1RA04219J-s001.pdf]

Electronic Supplementary Material (ESI) for RSC Advances.
This journal is © The Royal Society of Chemistry 2021

Electronic Supplementary Information (ESI)

Structural diversity in coordination compounds of cobalt, nickel and copper with *N*-alkylglycinates: crystallographic and ESR study in solid state

Darko Vušak,[a] Neven Smrečki,[a] Senada Muratović,[b] Dijana Žilić,[b] Biserka Prugovečki*[a] and Dubravka Matković-Čalogović[a]

[a.] *Department of Chemistry, Faculty of Science, University of Zagreb, Horvatovac 102a, HR-10000, Zagreb, Croatia.*
[b.] *Laboratory for Magnetic Resonances, Division of Physical Chemistry, Ruđer Bošković Institute, Bijenička 54, HR-10000 Zagreb, Croatia.*

***Corresponding author: biserka@chem.pmf.hr**

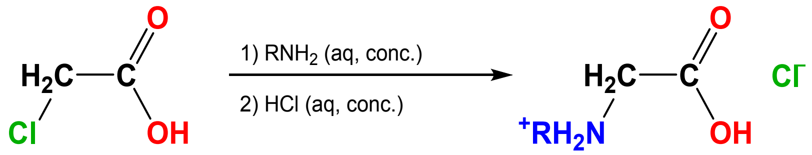

$H_2RGlyCl$ (R = Et, Pr)

Scheme S1. Prepration of *N*-ethylglycine-hydrochloride and *N*-propylglycine-hydrochloride by the aminolysis of chloroacetic acid.[40]

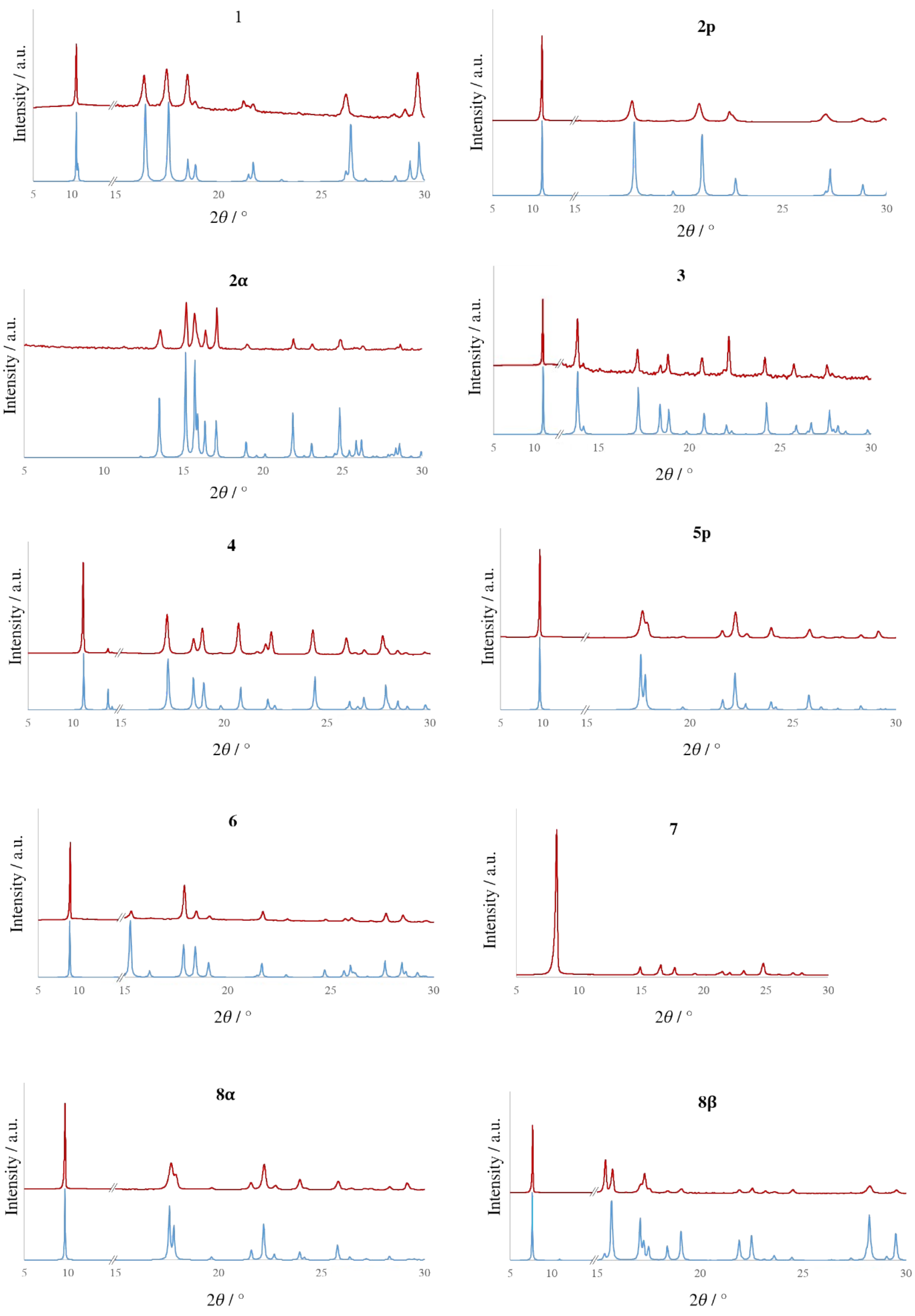

Figure S1. Experimental (red line) and calculated (blue line) powder pattern of the prepared compounds. Two parts of powder patterns separated with broken lines are not on the same intensity scale.

	Recrystallization of **2α**	Recrystallization of **8α**
H_2O	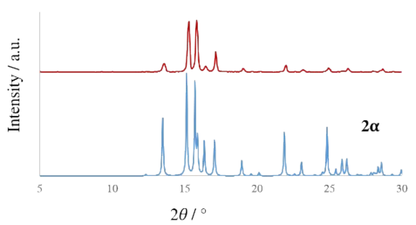	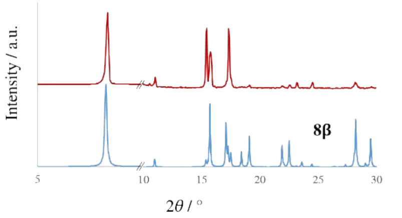
H_2O / CH_3OH	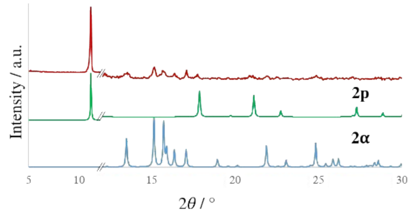	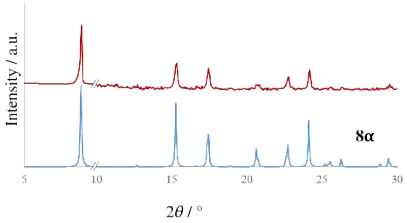
H_2O / CH_3CN	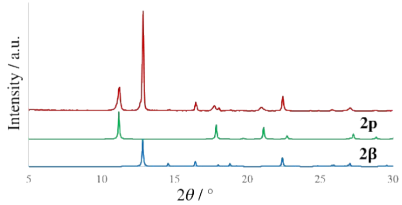	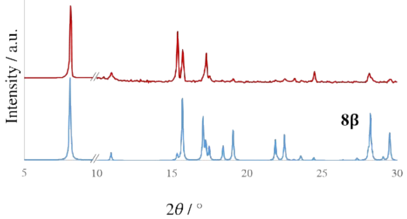
H_2O / $(CH_3)_2CO$	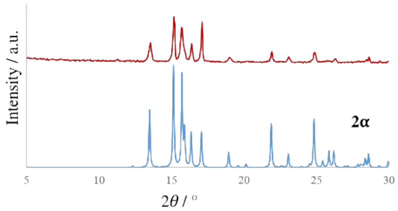	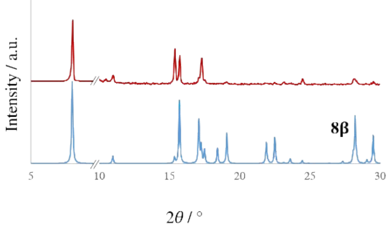
NH_4OAc(aq)	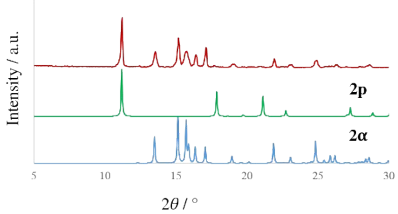	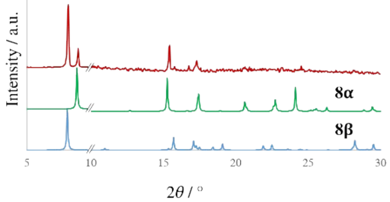
NH_3(aq)	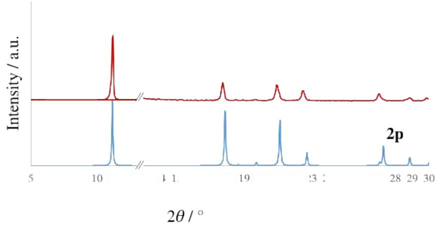	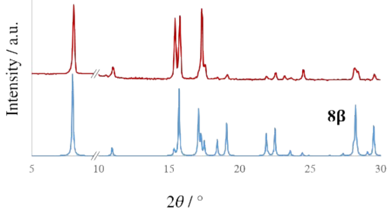

Figure S2. Experimental (red lines) powder patterns of crystals obtained after recrystallization of **2** and **8α** from different solutions compared with calculated powder patterns (blue and green lines).

2α

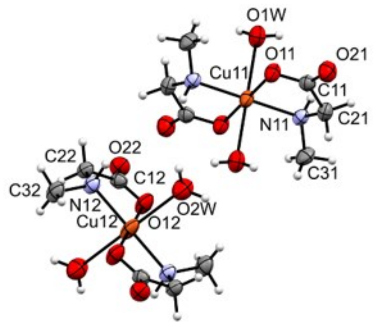

3

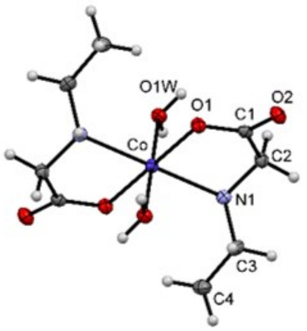

6

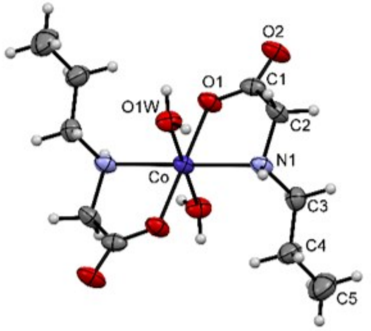

8β

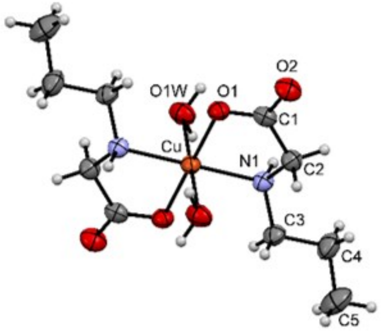

5p

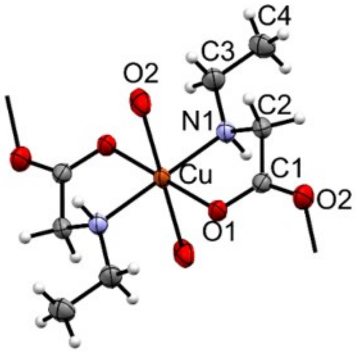

Figure S3. Molecular structures of coordination compounds **2α**, **3**, **6** and **8β**, and **5p.** Displacement ellipsoids of non-hydrogen atoms are drawn at the 50 % probability level.

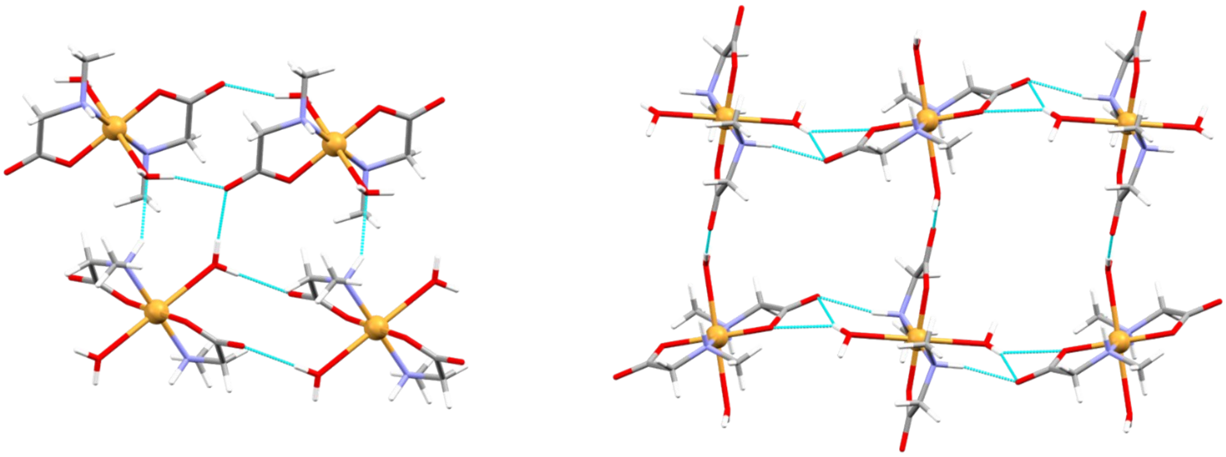

Figure S4. Hydrogen bond motifs in **2α**.

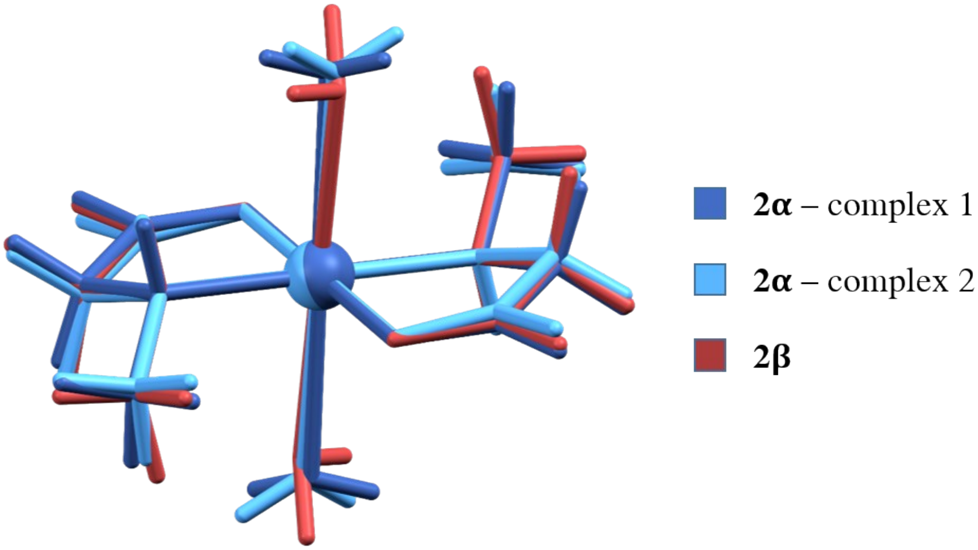

Figure S5. Overlapped complex molecules of compound [Cu(MeGly)$_2$(H$_2$O)$_2$] from monoclinic polymorph (**2β**), (CSD refcode POBDIT) and two symmetrically independent complexes in triclinic polymorph (**2α**). Molecules are overlapped over copper atoms and coordinated oxygen and nitrogen atoms.

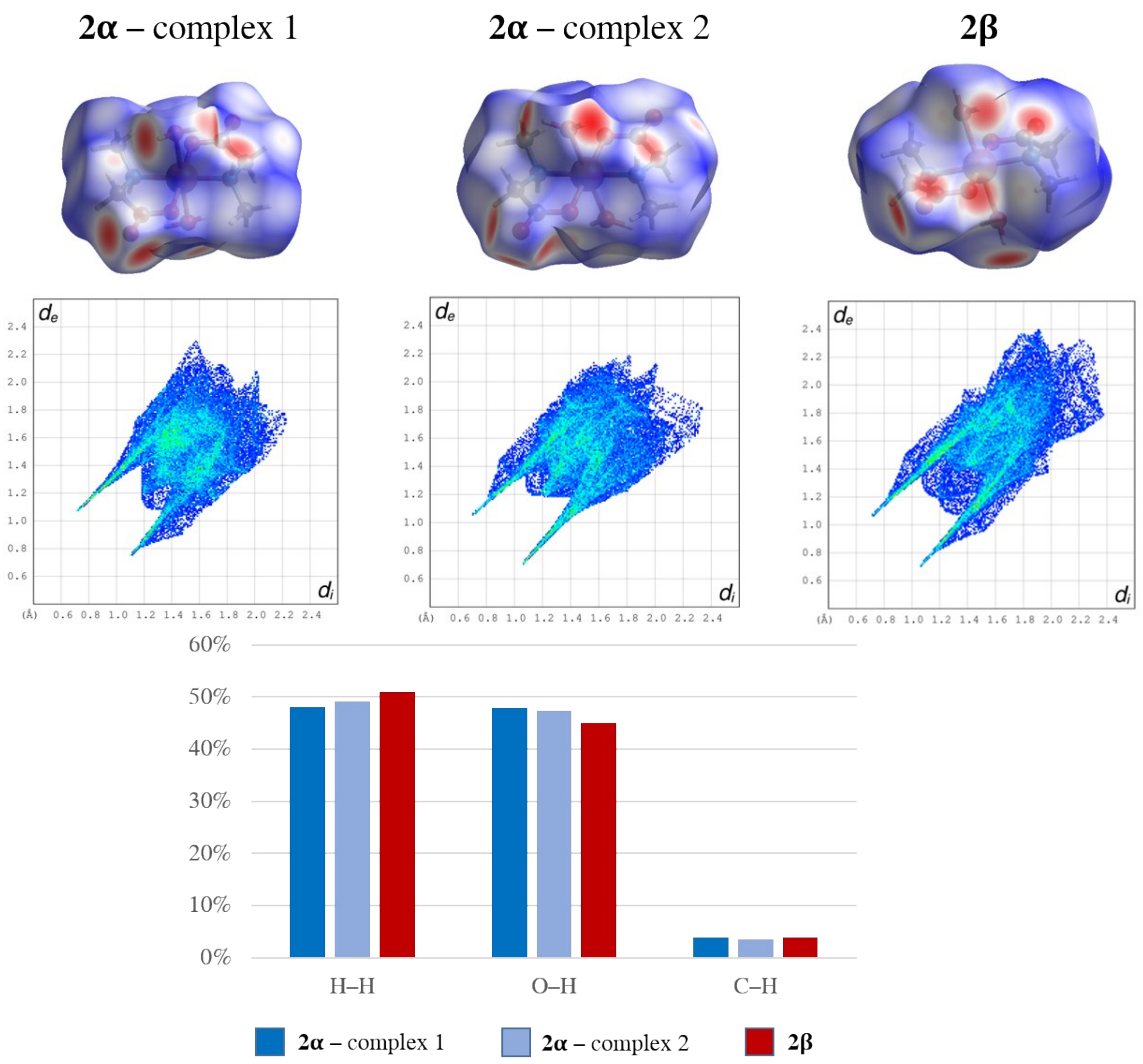

Figure S6. Hirshfeld fingerprint plot with decomposition of the dominant types of intermolecular contacts in monoclinic (**2β**) and triclinic (**2α**) polymorph of $[Cu(MeGly)_2(H_2O)_2]$. Two symmetrically independent complexes in **2α** are also decomposed. Intermolecular contacts: H–H (51.0% in **2β**; 48.1% in **2α** – complex 1; 49.1% in **2α** – complex 2), O–H (45.0% in **2β**; 47.9% in **2α** – complex 1; 47.3% in **2α** – complex 2), C–H (3.9% in **2β**; 4.0% in **2α** – complex 1; 3.6% in **2α** – complex 2).

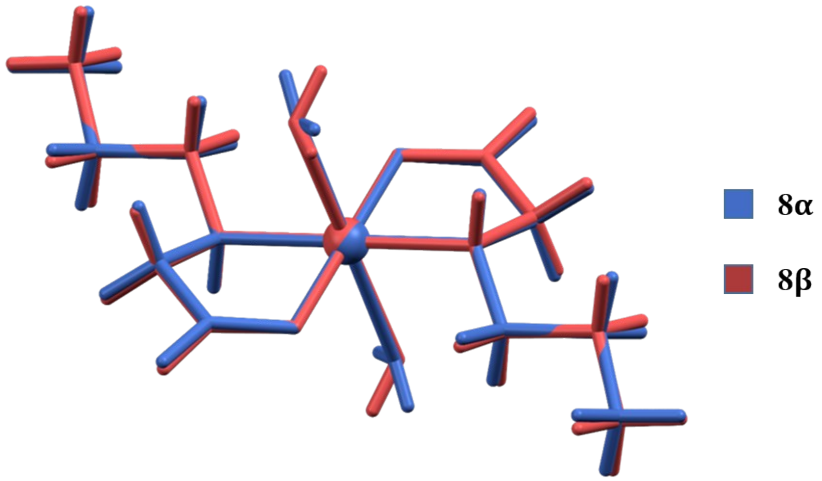

Figure S7. Overlapped coordination compounds of **8α** (dark blue) and **8β** (red). Molecules are overlapped over copper atoms and coordinated oxygen and nitrogen atoms.

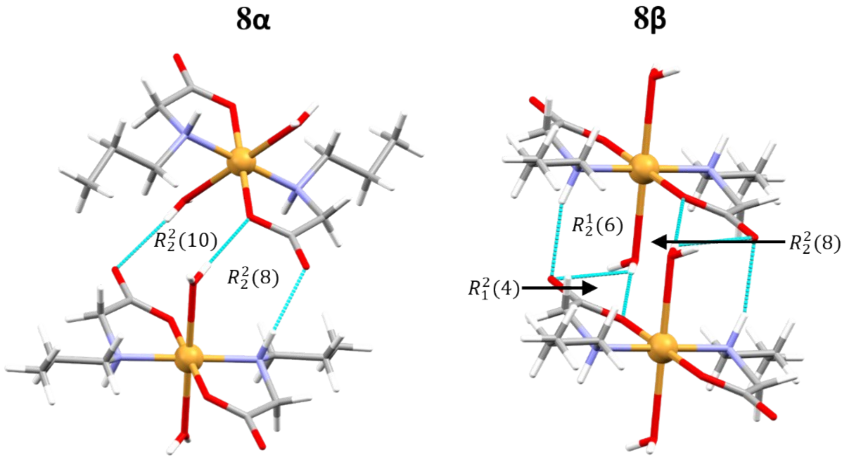

Figure S8. Two coordination compounds molecules interconnected through hydrogen bonds in **8α** and **8β**, with graph-set notation. Hydrogen bonds are shown as light blue lines.

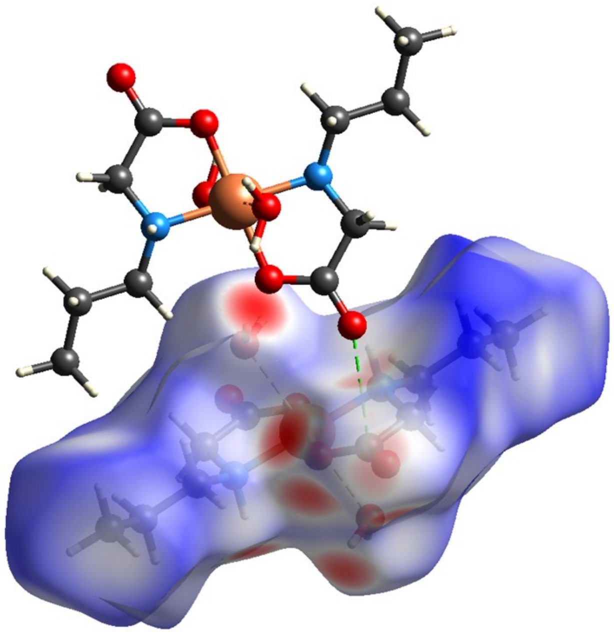

Figure S9. C–O···$C_{carboxylate}$ close contact (green dashed line) between two coordination compounds species in **8β**.

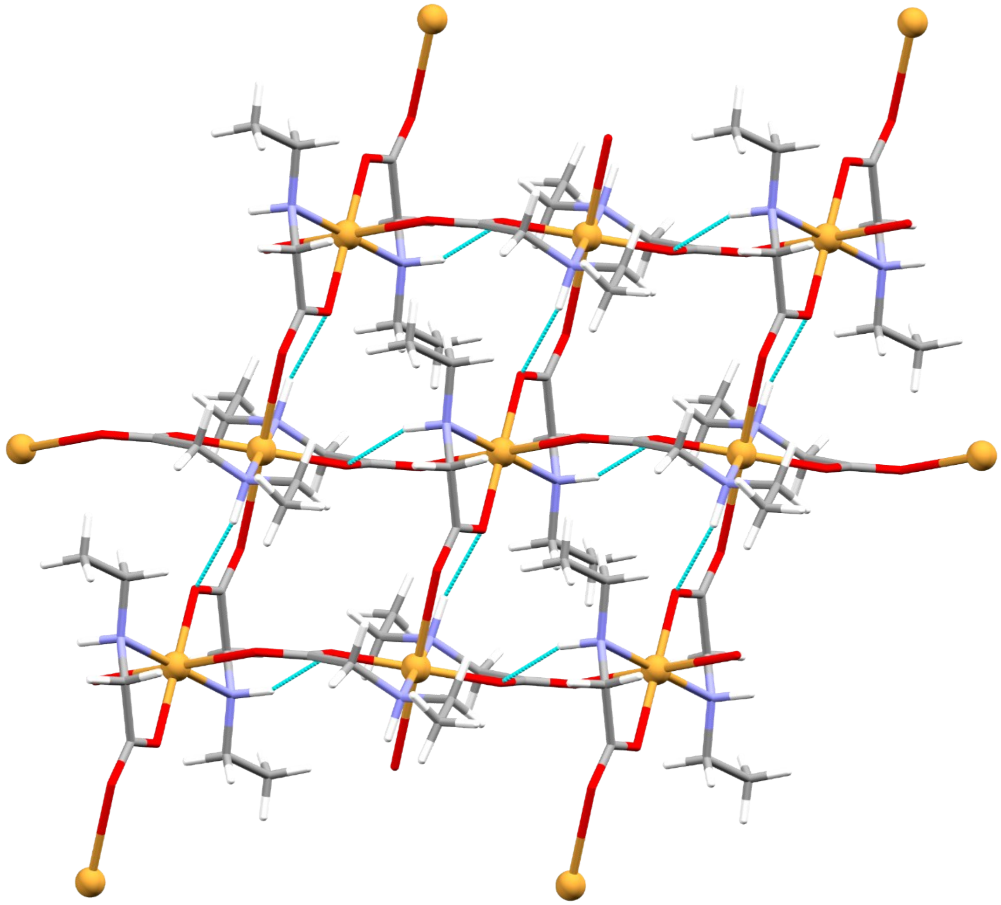

Figure S10. Intramolecular N–H···O hydrogen bonds within the polymeric network in **5p**. Hydrogen bonds are shown as light blue lines.

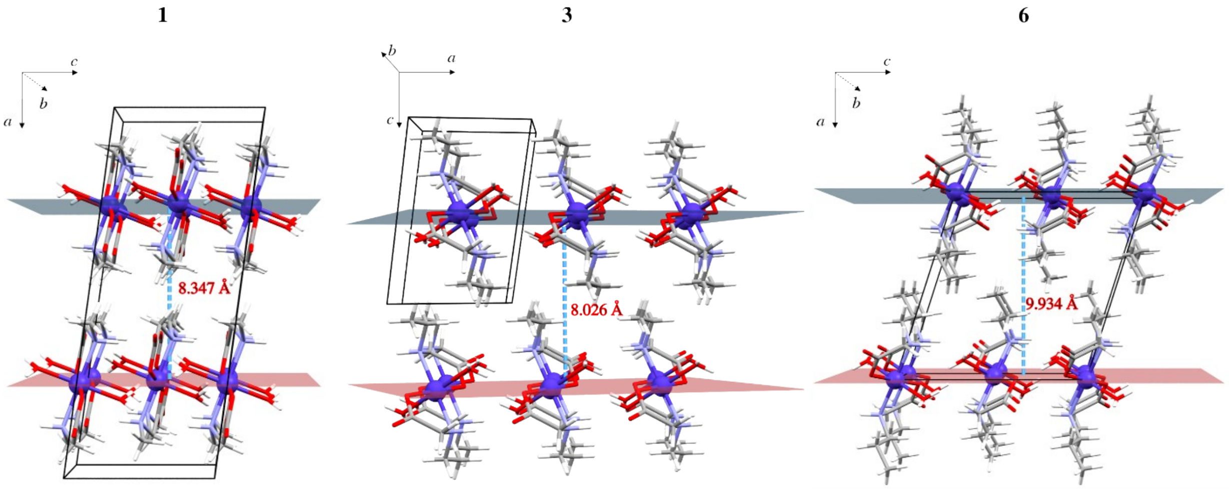

Figure S11. Distances between hydrogen-bonded 2D layers in compounds **1**, **3** and **6** in perspective view along crystallographic *b*-axis.

Table S1. Crystallographic data for coordination compounds [Co(MeGly)$_2$(H$_2$O)$_2$] (**1**), [{Co(MeGly)$_2$}$_2$(μ-OH)$_2$]·2H$_2$O (**1d**), [Cu(MeGly)$_2$(H$_2$O)$_2$] (**2α)** and [Cu(μ-MeGly)$_2$]$_n$ (**2p**).

Compound	**1**	**1d**	**2α**	**2p**
Formula	$C_6H_{16}CoN_2O_6$	$C_{12}H_{30}Co_2N_4O_{12}$	$C_6H_{16}CuN_2O_6$	$C_6H_{12}CuN_2O_4$
Formula weight	271.14	540.26	275.76	239.72
Space group	*C* 2/*c*	*P bca*	$P\bar{1}$	*P* 2$_1$/*c*
a/Å	16.8488(8)	15.5085(8)	7.0311(10)	7.9367(1)
b/Å	9.3909(4)	14.6844(6)	7.4825(11)	5.9953(1)
c/Å	6.8017(3)	18.0637(9)	10.6021(10)	9.0214(2)
α/°	90	90	78.541(10)	90
β/°	97.788(5)	90	89.938(10)	90.522(2)
γ/°	90	90	78.408(13)	90
V/Å^3	1066.28(8)	4113.7(3)	535.07(12)	429.247(13)
D_{calc}/g cm^{-3}	1.689	1.745	1.712	1.855
μ/mm^{-1}	1.622	1.681	2.053	3.531
F(000)	564	2240	286	246
θ range/°	4.3–27.0	2.3–26.0	4.4–27.0	5.4–74.9
T/K	295	295	295	295
Radiation wavelength	0.71073	0.71073	0.71073	1.54184
Range of *h*, *k*, *l*	-21–21, -11–11, -8–8	-19–19, -18–12, -11–22	-8–8, -9–9, -7–13	-9–9, -7–7, -10–11
Reflections collected	3485	18569	4095	10596
Independent reflections	3485	4042	2321	876
Observed reflections ($I \geq 2\sigma$)	2600	2961	2089	826
R_{int}	0.033	0.074	0.020	0.034
R^a, $wR^b[I \geq 2\sigma(I)]$	0.0264, 0.0780	0.0424, 0.0909	0.0292, 0.0825	0.0303, 0.0907
Goodness-of-fit, S^c	0.96	1.02	1.09	1.09
No. of parameters	80	296	157	61
$\Delta\rho_{min}$, $\Delta\rho_{max}$ (e Å^{-3})	-0.23, 0.39	-0.49, 0.48	-0.36, 0.36	-0.49, 0.57
CCDC no.	2069284	2069285	2069291	2084505

$^a R = \Sigma||F_o| - |F_c||/\Sigma|F_o|$; $^b wR = [\Sigma(F_o^2 - F_c^2)^2/\Sigma w(F_o^2)^2]^{1/2}$; $^c S = \Sigma[w(F_o^2 - F_c^2)^2/(N_{obs} - N_{param})]^{1/2}$

Table S2. Crystallographic data for coordination compounds [Co(EtGly)$_2$(H$_2$O)$_2$] (**3**) and [Ni(EtGly)$_2$(H$_2$O)$_2$] (**4**).

Compound	**3**		**4**	
Formula	$C_8H_{20}CoN_2O_6$	$C_8H_{20}CoN_2O_6$	$C_8H_{20}N_2NiO_6$	$C_8H_{20}N_2NiO_6$
Formula weight	299.19	299.19	298.95	298.95
Space group	$P\bar{1}$	$P\bar{1}$	$P\bar{1}$	$P\bar{1}$
a/Å	5.4524(2)	5.4717(3)	5.4231(3)	5.44882(8)
b/Å	7.1733(3)	7.1941(4)	7.1382(4)	7.15455(10)
c/Å	8.7708(3)	8.8468(4)	8.7608(4)	8.83552(11)
α/°	113.139(4)	113.095(4)	113.806(5)	113.6920(12)
β/°	91.989(3)	92.008(4)	91.508(4)	91.6006(11)
γ/°	106.635(3)	106.385(5)	106.771(5)	106.5605(12)
V/Å^3	298.00(2)	303.11(3)	293.12(3)	298.320(8)
D_{calc}/g cm^{-3}	1.667	1.639	1.694	1.664
μ/mm^{-1}	1.459	11.317	1.675	2.568
F(000)	157	157	158	158
θ range/°	4.2−27.0	5.5−72.4	4.3−27.0	5.5−79.6
T/K	150	295	150	295
Radiation wavelength	0.71073	1.54184	0.71073	1.54184
Range of h, k, l	-6−6, -9−9, -11−11	-6−6, -8−8, -10−10	-6−6, -9−9, -11−11	-6−6, -9−8, -11−11
Reflections collected	4798	3012	4697	7113
Independent reflections	1298	1186	1272	1268
Observed reflections ($I \geq 2\sigma$)	1267	1186	1255	1249
R_{int}	0.030	0.051	0.022	0.023
R^a, wR^b[I≥2σ(I)]	0.0230, 0.0583	0.0484, 0.1261	0.0175, 0.0430	0.0221, 0.0599
Goodness-of-fit, S^c	1.07	1.06	1.09	1.11
No. of parameters	88	87	88	87
$\Delta\rho_{min}$, $\Delta\rho_{max}$ (e Å^{-3})	-0.31, 0.40	-0.77, 0.98	-0.30, 0.37	-0.36, 0.26
CCDC no.	2069290	2084504	2069289	2084506

Table S3. Crystallographic data for coordination compounds $[Cu(\mu\text{-EtGly})_2]_n$ (**5p**), $[Co(PrGly)_2(H_2O)_2]$ (**6**), and $[Cu(PrGly)_2(H_2O)_2]$ (**8α** and **8β**).

Compound	**5p**	**6**	**8α**	**8β**
Formula	$C_8H_{16}CuN_2O_4$	$C_{10}H_{24}CoN_2O_6$	$C_{10}H_{24}CuN_2O_6$	$C_{10}H_{24}CuN_2O_6$
Formula weight	267.77	327.24	331.85	331.85
Space group	$P\,2_1/c$	$P\,2_1/c$	$I\,2/a$	$P\,2_1/c$
a / Å	10.0467(8)	10.4296(13)	9.3725(4)	11.5413(6)
b / Å	6.3568(5)	7.2609(9)	7.3714(4)	11.2713(5)
c / Å	9.0286(9)	10.0987(10)	21.2849(7)	5.8447(2)
α / °	90	90	90	90
β / °	114.398(10)	107.734(13)	90.496(3)	93.037(4)
γ / °	90	90	90	90
V / Å^3	525.12(9)	728.42(16)	1470.49(11)	759.24(6)
D_{calc} / g cm^{-3}	1.694	1.492	1.499	1.452
μ / mm^{-1}	2.076	1.201	1.508	1.460
$F(000)$	278	346	700	350
θ range / °	4.5–26.5	4.1–26.0	4.3–27.0	4.2–29.0
T / K	295	293	295	295
Radiation wavelength	0.71073	0.71073	0.71073	0.71073
Range of h, k, l	-11–12, -7–7, -11–10	-12–12, -8–8, -12–12	-10–11, -9–4, -16–27	-15–15, -15–15, -7–4
Reflections collected	4536	2294	2865	7331
Independent reflections	1074	2294	1597	2003
Observed reflections ($I \geq 2\sigma$)	968	1435	1275	1714
R_{int}	0.024	0.051	0.023	0.027
R^a, $wR^b[I\geq 2\sigma(I)]$	0.0277, 0.0703	0.0379, 0.0823	0.0379, 0.0809	0.0298, 0.0835
Goodness-of-fit, S^c	1.05	0.86	1.07	1.07
No. of parameters	71	98	97	97
$\Delta\rho_{min}$, $\Delta\rho_{max}$ (e Å^{-3})	-0.20, 0.47	-0.33, 0.49	-0.31, 0.37	-0.94, 0.77
CCDC no.	2069288	2069287	2069286	2069292

$^a R = \Sigma||F_o| - |F_c||/\Sigma|F_o|$; $^b wR = [\Sigma(F_o^2 - F_c^2)^2/\Sigma w(F_o^2)^2]^{1/2}$; $^c S = \Sigma[w(F_o^2 - F_c^2)^2/(N_{obs} - N_{param})]^{1/2}$

Table S4. Thermogravimetric analyses data.

Compound	Water loss			Starting temperature of complex decomposition / °C
	T / °C	w(theor.) / %	w(exp.) / %	
[Co(MeGly)$_2$(H$_2$O)$_2$] (**1**)	110	13.29	13.37	300
[Ni(MeGly)$_2$(H$_2$O)$_2$] (**1c**)	140	13.31	13.56	320
[Cu(MeGly)$_2$(H$_2$O)$_2$] (**2α**)	90	13.07	13.51	210
[Co(EtGly)$_2$(H$_2$O)$_2$] (**3**)	110	12.04	12.06	310
[Ni(EtGly)$_2$(H$_2$O)$_2$] (**4**)	120	12.05	12.16	315
[Cu(μ-EtGly)$_2$]$_n$ (**5p**)	/	/	/	220
[Co(PrGly)$_2$(H$_2$O)$_2$] (**6**)	90	11.01	11.07	310
[Ni(EtGly)$_2$(H$_2$O)$_2$] (**7**)	110	11.02	10.75	280
[Cu(PrGly)$_2$(H$_2$O)$_2$] (**8α**)	90	10.86	10.85	205
[Cu(PrGly)$_2$(H$_2$O)$_2$] (**8β**)	95	10.86	10.69	195

Table S5. Selected bond lengths (Å) and angles (°) for monomeric compounds **1**, **2α**, **3**, **4**, **6**, **8α** and **8β**. * M = Co in compounds **1**, **3** and **6**; M = Ni in compounds **4** and **7**, M = Cu in compounds **2α**, **8α** and **8β**; # There are two independent halves of the copper complex molecules in **2α** and corresponding labels are M11, O11, N11, O21, O11W and M12, O12, N12, O22**,** O12W

	**1**	**2α#**	**3**	**4**	**6**	**8α**	**8β**
M–O1 and M–O1*i*	2.1091(17)	1.9497(15)<br>1.9637(16)	2.0779(11)	2.0503(10)	2.069(2)	1.9788(16)	1.9645(12)
M–N1 and M–N1*i*	2.125(2)	2.0151(15)<br>2.0043(15)	2.1731(12)	2.1198(11)	2.175(3)	2.0203(17)	2.0191(14)
M–O1W and M–O1W*i*	2.108(2)	2.6008(17)<br>2.6008(17)	2.1407(11)	2.0985(10)	2.162(3)	2.445(2)	2.5426(13)
O1–M–O1W and O1i–M–O1Wi	93.65(8)	91.14(6)<br>91.61(7)	84.36(4)	84.67(4)	88.12(10)	85.44(8)	83.24(4)
O1–M–N1 and O1*i*–M–N1*i*	100.29(7)	95.59(6)<br>96.16(6)	80.64(4)	82.12(4)	99.82(10)	83.60(7)	95.91(5)
O1–M–O1*i*	180	180<br>180	180	180	180	180	180
O1–M–O1W*i* and O1*i*–M–O1W	86.35(8)	88.86(6)<br>88.39(7)	95.64(4)	95.33(4)	91.88(10)	94.59(9)	96.76(4)
O1–M–N1*i* and O1*i*–M–N1	79.71(7)	95.59(6)<br>96.16(6)	99.36(4)	97.88(4)	80.18(10)	96.47(7)	84.10(5)
O1W–M–N1 and O1W*i*–M–N1*i*	90.81(8)	89.96(6)<br>89.07(7)	92.75(4)	92.19(4)	86.22(12)	86.77(8)	93.27(5)
O1W–M–O1W*i*	180	180<br>180	180	180	180	180	180
N1–M–N1*i*	180	180<br>180	180	180	180	180	180

i = 1/2-*x*,3/2-*y*,-*z* in **1**; *i* = 1-*x*,1-*y*,1-*z* in the first independent molecule and *i* = -*x*,-*y*,-*z* in the second independent molecule in **2α**; *i* = 2-*x*,2-*y*,1-*z* in **3**; *i* = 1-*x*,-*y*,-*z* in **4**; *i* = -*x*,-*y*,-*z* in **6**; *i* = 3/2-*x*,1/2-*y*,1/2-*z* in **7**; *i* = 1/2-*x*,1/2-*y*,1/2-*z* in **8α**; *i* = -*x*,-*y*,-*z* in **8β**.

Table S6. Selected bond lengths (Å) and angles (°) for dimeric compound **1d**.

Bond lengths (Å)		Bond angles (°)			
Co1–O1H	1.886(2)	O1H–Co1–O2H	82.29(10)	O1H–Co2–O2H	81.36(10)
Co1–O2H	1.898(2)	O1H–Co1–O11	173.56(9)	O1H–Co2–O13	90.51(9)
Co1–O11	1.904(3)	O1H–Co1–O12	92.78(10)	O1H–Co2–O14	90.64(10)
Co1–O12	1.896(2)	O1H–Co1–N11	90.89(11)	O1H–Co2–N13	173.13(12)
Co1–N11	1.951(3)	O1H–Co1–N12	93.96(11)	O1H–Co2–N14	92.21(11)
Co1–N12	1.973(2)	O2H–Co1–O11	92.74(10)	O2H–Co2–O13	91.03(10)
Co2–O1H	1.904(2)	O2H–Co1–O12	173.76(10)	O2H–Co2–O14	91.45(11)
Co2–O2H	1.916(3)	O2H–Co1–N11	95.80(10)	O2H–Co2–N13	92.81(12)
Co2–O13	1.897(2)	O2H–Co1–N12	90.82(10)	O2H–Co2–N14	172.95(11)
Co2–O14	1.893(2)	O11–Co1–O12	92.44(10)	O13–Co2–O14	177.41(12)
Co2–N13	1.967(3)	O11–Co1–N11	85.52(12)	O13–Co2–N13	85.94(11)
Co2–N14	1.964(3)	O11–Co1–N12	90.20(11)	O13–Co2–N14	91.92(12)
		O12–Co1–N11	88.07(10)	O14–Co2–N13	93.16(11)
		O12–Co1–N12	85.69(9)	O14–Co2–N14	85.71(12)
		N11–Co1–N12	172.27(12)	N13–Co2–N14	93.79(13)

Table S7. Selected bond lengths (Å) and angles (°) for coordination polymer **5p**.

Bond lengths (Å)	
Cu–O1 and Cu–O1^{i}	1.9724(17)
Cu–O2 and Cu–O2^{i}	2.5228(18)
Cu–N1 and Cu–N1^{i}	2.004(2)
Bond angles (°)	
O1–Cu–O2	91.85(6)
O1–Cu–N1	96.70(7)
O1–Cu–O1^{i}	180
O1–Cu–O2^{i}	88.15(6)
O1–Cu–N1^{i}	83.30(7)
O2–Cu–N1	92.55(7)
O1^{i}–Cu–O2	88.15(6)
O2–Cu–O2^{i}	180
O1–Cu–N1^{i}	87.45(7)
O1^{i}–Cu–N1	83.30(7)
O2^{i}–Cu–N1	87.45(7)
N1–Cu–N1^{i}	180
O1^{i}–Cu–N2^{i}	91.85(6)
O1^{i}–Cu–N1^{i}	96.70(7)
O2^{i}–Cu–N1^{i}	92.55(7)

Table S8. Geometry of intermolecular hydrogen bonds (Å, °) for monomeric compounds **1**, **2α**, **3**, **4**, **6**, **8α** and **8β**.

	D–H···A	D–H (Å)	H···A (Å)	D···A (Å)	D–H···A(°)
**1**	N1–H1···O2[a]	0.98	2.06	2.970(3)	154
	O1W–H1WA···O1[a]	0.84(3)	1.93(3)	2.762(3)	172(3)
	O1W–H1WB···O2[b]	0.83(3)	1.90(3)	2.721(3)	170(3)
**2α**	O1W–H1WA···O21[c]	0.846(16)	1.914(16)	2.736(2)	163(3)
	O1W–H1WB···O12[d]	0.85(3)	2.44(3)	3.159(2)	143(3)
	O1W–H1WB···O22[d]	0.85(3)	2.15(3)	2.955(2)	159(3)
	O2W–H2WA···O22[e]	0.846(17)	2.013(17)	2.854(3)	173(3)
	O2W–H2WB···O21	0.844(17)	1.938(16)	2.781(3)	176.9(17)
	N11–H11···O22[d]	0.98	2.10	3.066(2)	169
	N12–H12···O1W[f]	0.98	2.19	3.063(2)	147
**3**	N1–H1···O2[g]	1.00	2.20	3.0688(17)	145
	O1W–H1WA···O2[a]	0.838(18)	1.900(18)	2.7366(17)	177(2)
	O1W–H1WB···O1[b]	0.841(15)	1.969(15)	2.8082(15)	176(2)
**4**	N1–H1···O2[g]	1.00	2.21	3.0749(16)	144
	O1W–H1WA···O2[a]	0.835(16)	1.905(16)	2.7388(15)	176.9(17)
	O1W–H1WB···O1[b]	0.841(13)	1.978(13)	2.8172(14)	176.0(14)
**6**	N1–H1···O2[j]	0.98	2.03	3.003(4)	175
	O1W–H1WA···O2[j]	0.84(3)	2.02(3)	2.799(4)	155(3)
	O1W–H1WB···O1[h]	0.83(3)	1.93(2)	2.749(4)	170(4)
**8α**	N1–H1···O2[k]	0.98	2.12	3.052(3)	158
	O1W–H1WA···O2[l]	0.74(3)	2.17(3)	2.899(3)	173(3)
	O1W–H1WB···O1[m]	0.72(3)	2.15(3)	2.862(3)	174(3)
**8β**	N1–H1···O2[j]	0.98	2.58	3.223(2)	123
	N1–H1···O2[n]	0.98	2.19	3.0722(18)	148
	O1W–H1WA···O1[j]	0.838(13)	2.102(15)	2.9164(18)	164(2)
	O1W–H1WB···O1[n]	0.84(2)	2.197(19)	3.0149(17)	164.9(19)
	O1W–H1WB···O2[n]	0.84(2)	2.54(2)	3.142(2)	129.5(19)

[a] 1/2-*x*,1/2+*y*,1/2-*z*; [b] *x*,1-*y*,1/2+*z*; [c] 1-*x*,-*y*,1-*z*; [d] *x*,*y*,1+*z*; [e] -*x*,1-*y*,-*z*; [f] -1+*x*,*y*,-1+*z*; [g] -1+*x*,*y*,*z*; [h] *x*,1+*y*,*z*; [i] 2-*x*,1-*y*,1-*z*; [j] -*x*,-1/2+*y*,-1/2-*z*; [k] 1/2+*x*,-*y*,*z*; [l] 1/2+*x*,1-*y*,*z*; [m] 1-*x*,1/2+*y*,1/2-*z*; [n] -*x*,-*y*,1-*z*

Table S9. Geometry of intra and intermolecular hydrogen bonds (Å, °) for dimeric compound **1d** and coordination polymer **5p.**

	D–H···A	D–H (Å)	H···A (Å)	D···A (Å)	D–H···A(°)
**1d**	O1W–H1WB···O24	0.84(5)	1.98(5)	2.812(6)	170(5)
	O1H–H1H···O23[a]	0.85(5)	1.89(2)	2.725(3)	167(3)
	O1W–H1WA···O12[b]	0.85(5)	2.36(3)	3.169(5)	161(4)
	O1W–H1WA···O22[b]	0.845(5)	2.36(2)	3.069(5)	141(4)
	O2H–H2H···O22[c]	0.84(5)	2.30(3)	3.088(3)	157(3)
	O2W–H2WA···O11	0.86(4)	2.14(5)	2.951(5)	159(3)
	O2W–H2WA···O21	0.86(4)	2.45(6)	3.041(5)	126(4)
	O2W–H2WB···O1W	0.85	2.02	2.824(6)	158
	N11–H11···O13	0.98	2.56	3.120(4)	116
	N11–H11···O22[c]	0.98	2.01	2.856(3)	144
	N12–H12··· O14	0.98	2.35	2.968(4)	120
	N12–H12···O23[a]	0.98	2.16	2.897(4)	131
	N13–H13···O2W[d]	0.98	2.29	3.223(5)	159
	N13–H13···O21[d]	0.98	2.48	3.109(4)	121
	N14–H14···O21[d]	0.98	2.24	3.039(4)	138
**5p**	N1–H1···O1[e]	0.98	2.09	3.002(2)	153

[a]1/2-*x*,-1/2+*y*,*z*; [b]-1/2+*x*,*y*,1/2-*z*; [c] 1/2-*x*,1/2+*y*,*z*; [d] *x*,3/2-*y*,1/2+*z*; [e]*x*,1/2-*y*,1/2+*z*

Table S10. Packing index of *N*-alkylglycinate complexes with hydrogen-bonded 2D layers. All data were calculated from room temperature data.

Compound	Packing index / %
**1**	72.9
**1d**	72.6
**1c**	72.0
**2α**	72.2
**2β**	68.7
**2p**	74.9
**3**	75.0
**4**	74.9
**5p**	72.8
**6**	70.7
**8α**	69.2
**8β**	67.4
